# Efficacy of combination therapy of vitamin D and bisphosphonates in the treatment of postmenopausal osteoporosis: a systematic review and meta-analysis

**DOI:** 10.3389/fphar.2024.1422062

**Published:** 2024-11-21

**Authors:** Yuangui Yang, Mingyue Yang, Xuanyi Su, Feibin Xie

**Affiliations:** ^1^ School of Clinical Medicine, Xiamen University, Xiamen, China; ^2^ School of Clinical Medicine, Fujian Medical University, Fuzhou, China; ^3^ Department of Orthopedic Trauma, Zhongshan Hospital of Xiamen University, School of Medicine, Xiamen University, Xiamen, China

**Keywords:** postmenopausal osteoporosis, combination treatment, vitamin D, diphosphonates, monotherapy

## Abstract

**Objective:**

There is currently no consensus on whether the combination therapy of Vitamin D (VitD) and bisphosphonates offers superior efficacy compared to monotherapy in the treatment of postmenopausal osteoporosis. The aim of this study is to conduct a meta-analysis of recent relevant research to synthesize the available evidence and further investigate whether the combined use of VitD and bisphosphonates is superior to monotherapy in treating osteoporosis in postmenopausal women.

**Methods and results:**

We systematically searched PubMed, EMBASE, the Cochrane Library, and Web of Science for randomized controlled trials (RCTs) comparing the effects of monotherapy with VitD or bisphosphonates *versus* their combination therapy in the treatment of postmenopausal osteoporosis, up to 1 February 2024. The articles were independently screened and relevant data were extracted by two investigators. The changes in mean values and percentage changes for bone resorption markers, bone formation markers, bone mineral density, and bone mineral metabolism markers were expressed using the standardized mean difference (SMD) and 95% confidence intervals (CI). Heterogeneity was quantitatively described using the I^2^ test. Subsequently, sensitivity analyses were performed for data with significant heterogeneity. Subgroup analyses were conducted based on the type of monotherapy used, and potential publication bias was assessed. The analysis revealed that the combination of VitD and bisphosphonates demonstrated a more pronounced effect in increasing alkaline phosphatase (ALP), 25-hydroxyvitamin D (25-OH-VD), and serum calcium (sCa) levels, as well as in decreasing levels of serum bone-specific alkaline phosphatase (sBALP), serum C-terminal telopeptide of type I collagen (sCTX), and urinary N-telopeptide of type I collagen (UriNTX) compared to the monotherapy group. However, the combination of VitD and bisphosphonates did not show a significant advantage over monotherapy in terms of improving osteocalcin levels. The differences in the mean changes in osteocalcin, UriNTX, and sCa, as well as the percentage changes in parathyroid hormone (PTH) were not statistically significant (*p* > 0.05).

**Conclusion:**

The meta-analysis suggests that compared to monotherapy, the combination therapy of VitD and bisphosphonates exhibits a more favorable effect on bone mineral density and bone calcium metabolism-related markers in the treatment of postmenopausal osteoporosis.

**Systematic Review Registration:**

https://www.crd.york.ac.uk/PROSPERO/PROSPERO

## 1 Introduction

Osteoporosis, as one of the common diseases among older individuals, has a global prevalence of approximately 18.3% (Wu et al., 2024), with about 30% of postmenopausal women affected by osteoporosis (Vilaca et al., 2022). The disease is characterized by reduced bone mass and deteriorated bone tissue structure (Zhang et al., 2023). This not only leads to increased bone fragility and a higher risk of fractures, with the lifetime fracture risk for patients reaching up to 40% (Rachner et al., 2011), but also significantly diminishes the quality of life for elderly patients, potentially leading to disability and even death. Therefore, it is particularly important to explore the optimal treatment strategies for osteoporosis.

To date, the pharmacological treatment options for osteoporosis primarily fall into two main categories: anabolic agents that promote bone formation and antiresorptive agents that inhibit bone resorption. The former’s main component is active vitamin D, with common medications including alfacalcidol and calcitriol, which directly affect the balance and metabolism of calcium and phosphorus in the body (Lips and van Schoor, 2011). Antiresorptive agents primarily consist of bisphosphonates, such as alendronate sodium and risedronate sodium. These medications effectively reduce the risk of fractures in areas like the spine and hip by disrupting the precursor differentiation function of osteoclasts (McClung et al., 2013; Vannala et al., 2020). Patricia Barrionuevo and colleagues conducted a network meta-analysis that demonstrated a significant reduction in the probability of fractures among postmenopausal women with osteoporosis treated with alendronate sodium compared to those in the placebo group (Barrionuevo et al., 2019). The effectiveness of monotherapy for osteoporosis has been established, as noted by P Lips, who pointed out that oral Vitamin D (VitD) can improve bone mineral density and bone metabolic markers, promoting calcification of bone tissue to treat osteoporosis (Lips, 2001).

Combination therapy has been increasingly used in the treatment of osteoporosis, particularly in postmenopausal women (Saul and Drake, 2021). However, there is still no consensus on whether combination therapy is superior to monotherapy for osteoporosis (Pinkerton and Dalkin, 2007). In an expert consensus document (Huang et al., 2023) regarding the prevention and treatment of osteoporosis, the combination therapy of bisphosphonates and Vitamin D in the treatment of postmenopausal osteoporosis has been affirmed, but there is still a scarcity of robust evidence-based medical evidence to assess the safety and effectiveness of such combined therapy. Therefore, this study holds certain guidance significance and clinical value for the pharmaceutical treatment of postmenopausal osteoporosis. Research conducted by Z. L. Zhang has demonstrated that the use of combined alendronate sodium and vitamin D3 tablets can more effectively treat postmenopausal women with osteoporosis and alleviate symptoms of the disease (Zhang et al., 2015). However, research conducted by Joel S. Finkelstein and colleagues suggests that the combined use of teriparatide and alendronate sodium did not show superior efficacy compared to the use of teriparatide alone (Finkelstein et al., 2010). Compared to analyzing a single study, a systematic review synthesizes all available evidence, providing a more comprehensive analysis of therapeutic efficacy.

The purpose of this study is to conduct a meta-analysis of recent relevant research to synthesize and analyze various clinical diagnostic and prognostic indicators of osteoporosis, such as bone mineral density (BMD) at different sites (lumbar BMD [LBMD], femoral neck BMD [ftroBMD], femur BMD [fBMD], and total hip BMD [ThipBMD]), bone resorption markers (Carboxy terminal collagen crosslinks in serum [sCTX], urinary N-terminal cross-linked telopeptides of type I collagen [UriNTX]), bone formation markers (osteocalcin [OC], alkaline phosphatase [ALP], serum bone-specific alkaline phosphatase [sBALP]), bone calcium metabolism indicators (parathyroid hormone [PTH], serum calcium [sCa], 25-hydroxyvitamin D [25-OH-VD]) and safety metrics, to further investigate whether the combined treatment of VitD and bisphosphonates is superior to monotherapy in treating postmenopausal osteoporotic women. This research aims to provide a scientific basis for the effective clinical treatment of osteoporosis.

## 2 Materials and methods

The study adhered to the Preferred Reporting Items for Systematic Reviews and Meta-Analyses (PRISMA) statement (Page et al., 2021). A formal protocol was established and registered in advance on the PROSPERO platform of systematic review and meta-analysis protocols. (Registration number: CRD42023384638).

### 2.1 Literature search selection criteria

The systematic search conducted by two researchers (YG Y and MY Y) across authoritative databases, including PubMed, EMBASE, the Cochrane Library, and Web of Science, aimed to retrieve randomized controlled trials (RCTs) comparing monotherapy with combination therapy of VitD and bisphosphonates in the treatment of postmenopausal osteoporosis up to 1 February 2024. The search was not restricted by language, ensuring a comprehensive literature review. The search strategy utilized a combination of Medical Subject Headings (MeSH) terms and free text words to enhance the sensitivity and specificity of the search. The key search terms included “Vitamin D,” “Diphosphonates,” “osteoporosis,” and other relevant terms. The detailed search strategy for the aforementioned databases is provided in the supplementary attachment.The two researchers (MY Y and XY S) began by reviewing the titles and abstracts of the identified literature. They documented the reasons for excluding certain studies. In cases where it was not possible to determine inclusion or exclusion based solely on the title and abstract, they proceeded to carefully examine the full text of the studies. When discrepancies arose between the two researchers during the screening process, a third researcher (YG Y) was consulted to resolve the disagreement through discussion. If the articles meet the aforementioned criteria, they will be selected for further analysis. The inclusion criteria were as follows: 1) Population:Postmenopausal women with osteoporosis; 2) Intervention: combination therapy of vitaminD (VitD3,alfacalcidol, calcitrol,etc.) and bisphosphonates (alendronate, zoledronateacid,etc.); 3) Comparison: Monotherapy of VitD or biphosphonate; 4) Outcome: bonemineral density (BMD), fracture incidence,etc.,; 5) Design: RCT. Subsequently, the third researcher (YG Y) conducted a summary of the screening results. Based on the established exclusive criteria, studies were excluded for the following reasons: 1) non-relevant study types; 2) studies with experimental groups limited to monotherapy; 3) duplicate publications; 4) animal studies; 5) study populations with conditions affecting bone density, such as HIV, cirrhosis, thalassemia, etc.; 6) absence of pertinent outcome measures; 7) research subjects with a history of organ transplantation, gastrectomy, or long-term glucocorticoid use.

### 2.2 Data extraction and quality assessment

For each article that met the inclusion criteria, two researchers (MY Y and XY S) independently extracted relevant data using a pre-designed data extraction form. Disagreements in data extraction were resolved through discussion or with the assistance of a third researcher (YG Y). The extracted data included:

a. Basic study information: the name of the first author, year of publication, and the region or country where the study was conducted.
b. Basic demographic information of the study population: sample size, average age, concomitant treatments, and other baseline clinical characteristics.
c. Outcome measures: Femoral neck Bone mineral density (F BMD),Total-hip Bone mineral density (T hip BMD),Lumbar spine Bone mineral density (LBMD),25-hydroxy-vitamin D (25-OH-VD),Femoral trochanter Bone mineral density (f trochanterBMD),Urinary N-terminal cross-linked telopeptides of type Ⅰ collagen (Uri NTX),Osteocalcin (OC), sBALP, Carboxy terminal collagen crosslinks in serum (sCTX),Calcium in serum (sCa),Parathyroid hormone (PTH),Alkaline phosphatase (ALP). The assessment of the risk of bias in the included literature was independently conducted by two researchers, using the ROB 1.0 tool embedded in Review Manager 5.4 to evaluate the risk of bias in the included literature. The following items were assessed: 1) random sequence generation; 2) allocation concealment; 3) blinding of participants and personnel; 4) blinding of outcome assessment; 5) incomplete outcome data; 6) selective reporting or publication bias; 7) other bias. Each item of bias was evaluated, with “low risk” indicating a low risk of bias, “high risk” indicating a high risk of bias, and “unclear risk” indicating a medium risk of bias. We did not assign an overall quality rating to the literature.

### 2.3 Statistical analysis

The primary outcomes for postmenopausal osteoporosis patients receiving combination or monotherapy were the mean changes or percentage changes in bone mineral density, bone resorption markers, bone formation markers, and bone calcium metabolic markers. These outcomes were represented using the standard mean difference (SMD) and its 95% confidence interval (CI). Initially, the heterogeneity among studies was quantitatively described using the *I* ^*2*^ test. A *p*-value less than 0.05 was considered to indicate statistically significant differences. When the I^2^ value was less than 50% and the *p*-value was greater than 0.05, indicating low heterogeneity, the fixed-effect model was used for data synthesis. Otherwise, the random-effects model was applied. Secondly, for data showing significant heterogeneity, sensitivity analyses were conducted to identify the sources of heterogeneity and to assess whether they influenced the robustness of the results. Additionally, if the monotherapy group involved different types of monotherapy, subgroup analyses were conducted based on the types of monotherapy used in the monotherapy groups, followed by an analysis of statistical heterogeneity within each subgroup. Finally, when the number of included studies was 10 or more, publication bias was assessed using funnel plots and Egger’s test. If the funnel plot was symmetrical or the *p*-value was greater than or equal to 0.05, there was no significant publication bias. Conversely, if the funnel plot was asymmetrical or the *p*-value was less than 0.05, it indicated the presence of significant publication bias. In cases where significant publication bias was identified, the “trim and fill” algorithm was employed to correct for it. All statistical analyses were performed using R (version 4.3.2), with the primary packages involved being the ‘meta’ package (version 7.0–0). The confidence of evidence would be assessed by GRADE system, the results of assessment would be presented along with the pooling estimations (Inc, 2021) (Table 1).

**TABLE 1:** Table of GRADE.

Certainty assessment							No of patients		Effect		
No of studies	Study design	Risk of bias	Inconsistency	Indirectness	Imprecision	Other considerations	Dual VD-BP	Mono VD or BP	Relative (95% CI)	Absolute (95% CI)	Certainty
ftroBMD											
8	randomised trials	not serious	very serious^a^	not serious	not serious	none	2426	1475	-	SMD 2.57 SD higher (0.82 higher to 4.31 higher)	⊕⊕○○ Low^a^
LBMD											
22	randomised trials	not serious	very serious^a^	not serious	not serious	none	3239	2283	-	SMD 3.02 SD higher (1.61 higher to 4.42 higher)	⊕⊕○○ Low^a^
fBMD											
15	randomised trials	not serious	very serious^a^	not serious	not serious	none	2701	1734	-	SMD 1.93 SD higher (0.72 higher to 3.15 higher)	⊕⊕○○ Low^a^
ThipBMD											
10	randomised trials	not serious	very serious^a^	not serious	not serious	none	2675	1716	-	SMD 1.68 SD higher (0.3 higher to 3.06 higher)	⊕⊕○○ Low^a^
sCTX abs											
7	randomised trials	not serious	very serious^a^	not serious	not serious	none	332	290	-	SMD 1.22 SD lower (1.9 lower to 0.54 lower)	⊕⊕○○ Low^a^
sCTX per											
6	randomised trials	not serious	very serious^a^	not serious	not serious	none	2204	1224	-	SMD 1.45 SD lower (2.52 lower to 0.38 lower)	⊕⊕○○ Low^a^
UriNTX abs											
1	randomised trials	not serious	not serious	not serious	not serious	none	140	139	-	SMD 0.23 SD lower (0.47 lower to 0.01 higher)	⊕⊕⊕⊕ High
UniNTX per											
9	randomised trials	not serious	very serious^a^	not serious	not serious	none	1308	1246	-	SMD 1.74 SD lower (3.29 lower to 0.2 lower)	⊕⊕○○ Low^a^
sCa abs											
5	randomised trials	not serious	very serious^a^	not serious	not serious	none	262	272	-	SMD 0.94 SD higher (0.4 lower to 2.27 higher)	⊕⊕○○ Low^a^
sCa per											
1	randomised trials	not serious	not serious	not serious	not serious	none	90	90	-	SMD 2.99 SD higher (2.56 higher to 3.41 higher)	⊕⊕⊕⊕ High
25-OH-VD abs											
4	randomised trials	not serious	very serious^a^	not serious	not serious	none	584	571	-	SMD 1.61 SD higher (0.51 higher to 2.7 higher)	⊕⊕○○ Low^a^
25-OH-VD per											
2	randomised trials	not serious	very serious^a^	not serious	not serious	none	127	109	-	SMD 1.37 SD higher (0.54 higher to 2.19 higher)	⊕⊕○○ Low^a^
PTH abs											
4	randomised trials	not serious	serious^b^	not serious	not serious	none	280	274	-	SMD 0.74 SD lower (1.05 lower to 0.42 lower)	⊕⊕⊕○ Moderate^b^
PTH per											
4	randomised trials	not serious	very serious^a^	not serious	not serious	none	531	525	-	SMD 1.25 SD lower (3.19 lower to 0.69 higher)	⊕⊕○○ Low^a^
ALP											
5	randomised trials	not serious	very serious^a^	not serious	not serious	none	191	185	-	SMD 0.52 SD lower (1.02 lower to 0.02 lower)	⊕⊕○○ Low^a^
Osteocalcin abs											
6	randomised trials	not serious	very serious^a^	not serious	not serious	none	187	188	-	SMD 0.54 SD higher (2.76 lower to 3.84 higher)	⊕⊕○○ Low^a^
Osteocalcin per											
2	randomised trials	not serious	very serious^a^	not serious	not serious	none	380	350	-	SMD 1.81 SD lower (3.03 lower to 0.59 lower)	⊕⊕○○ Low^a^
sBALP abs											
3	randomised trials	not serious	serious^b^	not serious	not serious	none	495	492	-	SMD 0.64 SD lower (0.9 lower to 0.38 lower)	⊕⊕⊕○ Moderate^b^
sBALP per											
10	randomised trials	not serious	very serious^a^	not serious	not serious	publication bias strongly suspected^c^	1448	1344	-	SMD 1.38 SD lower (2.15 lower to 0.61 lower)	⊕○○○ Very low^a^ ^,^ ^c^
AE											
18	randomised trials	not serious	serious^b^	not serious	not serious	none	3811/6786 (56.2%)	2975/6786 (43.8%)	OR 1.03 (0.77–1.37)	1 more per 100 (from 6 fewer to 8 more)	⊕⊕⊕○ Moderate^b^
SAE											
7	randomised trials	not serious	not serious	not serious	not serious	none	2693/4411 (61.1%)	1718/4411 (38.9%)	OR 1.10 (0.93–1.30)	23 more per 1,000 (from 17 fewer to 64 more)	⊕⊕⊕⊕ High

CI: confidence interval; OR: odds ratio; SMD: standardised mean difference.

Explanations

^a^ I2 is very high.

^b^ I2 is high.

^c^ Egger P > 0.05.

## 3 Results

### 3.1 Literature search

**FIGURE 1:**
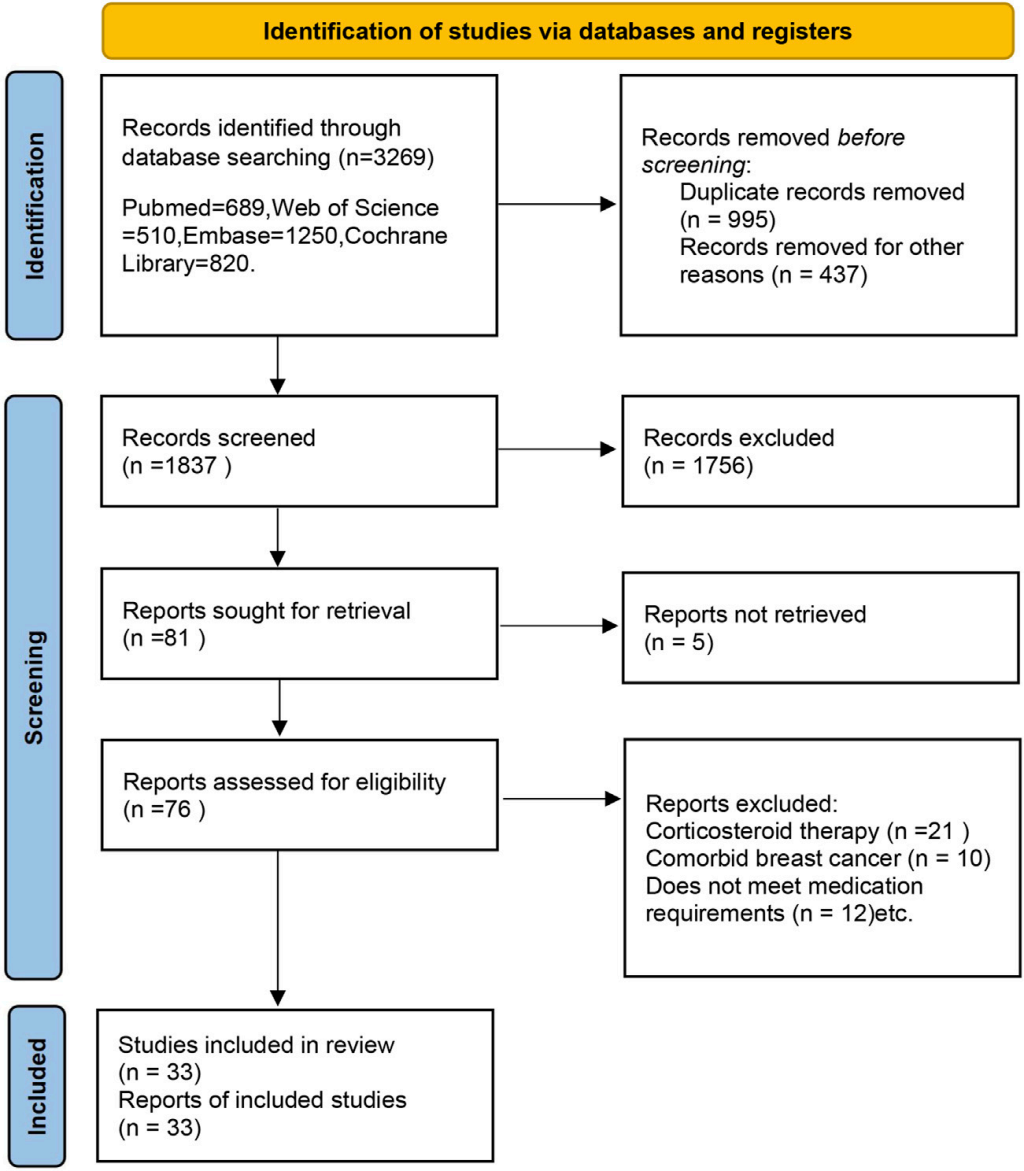
Flowchart of study selection in this meta-analysis.

The process of literature screening, study selection, and reasons for exclusion were described in a flowchart (Figure 1). Initially, we identified 3,268 records from our primary search (Supplementary Material S2). We then excluded 994 duplicates. After screening the titles and abstracts, 76 studies were deemed potentially eligible for inclusion. Upon full-text studies, 33 randomized controlled trials were ultimately included in the meta-analysis.

### 3.2 Characteristics of the included studies

Table 2 provides a summary of the baseline characteristics and medication details of the study populations included in this meta-analysis. The meta-analysis encompassed 33 studies published from 1997 to 2022, involving 8058 postmenopausal women with osteoporosis who received combination or monotherapy from Europe (17 studies), Asia (11 studies), and North America (five studies). The average age of the included participants ranged from 55 to 77 years old, with the sample size varying from 30 to 2860 individuals. In the 33 studies included in this meta-analysis, the experimental group utilized a combination of VitD and bisphosphonates. Beyond this, in nine studies, the control group only used bisphosphonates, while in 23 studies, the control group used VitD alone. The included studies in our analysis have largely adhered to the standard treatment guidelines for medication prescription: calcium supplementation is consistently maintained within the range of 0.5–1.0 g per day (g/d), Vitamin D supplementation is generally around 500 International Units per day (IU/d), Alendronate is commonly prescribed at a dose of 10 mg per day (mg/d), and Neridronate at a dosing range of 12.5–50 mg per month (mg/m). Other bisphosphonates also follow the standard treatment protocols. The duration of treatment varies from 3 months up to 48 months, with most treatments consisting of a complete cycle of either 12 or 24 months, as detailed in the Supplementary Table S2 Drug regimens of included studies. Additionally, one study featured control groups for both monotherapies, each using one of the drugs alone. The included participants were all postmenopausal women who did not suffer from HIV, liver cirrhosis, Eastern Mediterranean and other diseases affecting bone density. They also had no history of surgeries like organ transplantation or gastrectomy, nor did they have a history of long-term use of glucocorticoids. They were highly comparable.

**TABLE 2:** Characteristics of the included studies.

Reference	Country	Number	Age	Monotherapy	Combination therapy	Adjuvant baseline
Adami et al. (2008)	Italy	94	62.46 ± 6.49	Vitamin D 800IU/D	(Neridronate 50 mg/M or 12.5 mg/M or 25 mg/M) and vitamin D 800IU/D	Calcium 1g/D
Barone et al. (2007)	Italy	91	69.25 ± 6.92	ALN 10mg/D	1,25-D3 0.5μg/D and ALN 10mg/D	Calcium 1g/D
Bell et al. (2002)	USA	65	66.15 ± 8.77	Vitamin D 500IU/D	Alendronate 10mg/D and vitamin D 500IU/D	Calcium 0.5g/D
Braga et al. (2003)	Italy	78	64.55 ± 7.76	Vitamin D 400IU/D	Neridronate 50 mg/M and vitamin D 400IU/D	Calcium 0.5g/D
Cascella et al. (2005)	Italy	40	72.70 ± 5.19	Vitamin D 400IU/D	Neridronate 25 mg/M and vitamin D 400IU/D	Calcium 0.5g/D
Cesareo et al. (2015)	Italy	30	58.00 ± 5.02	Cholecalciferol 400 IU/D	Alendronate 10 mg/D and cholecalciferol 400 IU/D	Calcium 1g/D
Cheng et al. (2002)	China	56	63.95 ± 6.12	Vitamin D 1000IU/D	Alendronate sodium 10mg/D and vitamin D 1000IU/D	Calcium 0.6g/D
Dobnig et al. (2006)	Germany	56	67.98 ± 5.34	Vitamin D 400–800IU/D	(Risedronate 5mg/D or alendronate 10mg/D) and vitamin D 400–800IU/D	Calcium 1.0–1.2g/D
Dundar et al. (2009)	Turkey	61	60.35 ± 8.99	Vitamin D 400IU/D	Risedronate 5mg/D and vitamin D 400IU/D	Calcium 1g/D
Felsenberg et al. (2011)	Germany	279	73.67 ± 4.75	Alendronate 10mg/D	Alfacalcidol 1μg/D and alendronate 10mg/D	Calcium 0.5g/D
Frediani et al. (1998)	Italy	90	63.13 ± 6.51	Alendronate10mg/D or calcitriol 0.5μg/D	Alendronate 10mg/D and calcitriol 0.5μg/D	Calcium 0.5g/D
Greenspan et al. (2015)	USA	181	85.45 ± 5.22	Vitamin D 800IU/D	Zoledronate 5mg/D and vitamin D 800IU/D	Calcium 1.2g/D
Iwamoto and Sato (2014)	Japan	96	70.77 ± 9.19	Alendronate5mg/D or risedronate 2.5mg/D	Vitamin D 0.75μg/D and (alendronate 5mg/D or risedronate 2.5mg/D)	NR
Iwamoto et al. (2003)	Japan	40	71.15 ± 6.25	Etidronate 2800 mg/3M	Alfacalcidol 1μg/D and etidronate 2800 mg/3M	Calcium 0.8g/D
Karadag-Saygi et al. (2011)	Turkey	71	62.96 ± 7.43	Vitamin D 400IU/D	Risedronate 35 mg/W and vitamin D 400IU/D	Calcium 0.6g/D
Kim et al. (2014)	Korea	268	NR	ALN 10mg/D	Vitamin D 800IU/D	Calcium 0.2g/D
Leung et al. (2005)	China	65	67.00 ± 5.95	Vitamin D 400IU/D	Risedronate 5mg/D and vitamin D 400IU/D	Calcium 0.5g/D
Lyritis et al. (1997)	Athens	100	72.00 ± 0.41	Calcitriol R 0.4μg/D	Etidronate 400mg/D (20d) and calcitriol R 0.4μg/D	Calcium 0.5g/D
Masud et al. (1998)	Britain	47	66.29 ± 8.28	Etidronate 0.4g/D	Calcitriol 0.5 µg/D and etidronate 0.4g/D	Calcium 0.5g/D
Matsumoto et al. (2009)	Japan	674	71.55 ± 5.80	Vitamin D 200IU/D	Minodronate 1mg/D and vitamin D 200IU/D	Calcium 0.6g/D
McClung et al. (2009)	USA	160	53.54 ± 3.70	Vitamin D 400IU/D	Ibandronate 5mg/D and vitamin D 400IU/D	Calcium 0.5g/D
Nenonen et al. (2005)	Finland	148	53.50 ± 2.36	Vitamin D 200IU/D	Alendronate 5mg/D and vitamin D 200IU/D	Calcium 0.63g/D
Olmos et al. (2005)	Spain	140	68.04 ± 8.03	ALN 70 mg/W	Calcifediol 0.266 mg/W and ALN 70 mg/W	NR
Peng et al. (2022)	China	262	61.80 ± 5.99	Vitamin D3 200IU/D	Minodronate 1mg/D and vitamin D3 200IU/D	Calcium 0.5g/D
Popp et al. (2013)	Swit	107	76.80 ± 4.98	Vitamin D3	Zoledronate 5mg/Y and Vitamin D3	Calcium
Recker et al. (2006)	USA	717	66.80 ± 8.70	Alendronate 10mg/D	Alendronate 10mg/D and cholecalciferol 400IU/D	Calcium 0.5–0.6g/D
Recker et al. (2004)	USA	2860	67.00	Vitamin D 400IU/D	(Ibandronate 1 or 0.5 mg/3M) and vitamin D 400IU/D	Calcium 0.5g/D
Rhee et al. (2006)	Korea	199	62.04 ± 5.51	Alfacalcidol 1µg/D	Maxmarvil = Calcitriol 0.5µg/D and alendronate 5 mg	NR
Rossini et al. (2000)	Italy	124	63.01 ± 5.37	Vitamin D 440U/D	(alendronate 20 mg/W or alendronate 10mg/D for 1month/3month) and vitamin D 440U/D	Calcium 0.5g/D
Shiota et al. (2001)	Japan	40	61.70 ± 6.72	Alphacalcidol 0.5μg/D	Etidronate 200mg/D (2W) and alphacalcidol 0.5μg/D	Calcium 2g/D
Tanakol et al. (2007)	Turkey	79	56.88 ± 8.15	Vitamin D 400IU/D	Clodronic acid 400mg/D and vitamin D 400IU/D	Calcium 0.5g/D
Yan et al. (2009)	China	560	64.93 ± 6.18	Vitamin D 200IU/D	Alendronate 70 mg/W and vitamin D 200IU/D	Calcium 0.5g/D
You et al. (2011)	China	180	62.32 ± 6.86	Alfacalcidol 0.5μg/D	Alendronate 70 mg/2W and alfacalcidol 0.5μg/D	Calcium 0.6g/D and vitamin D3 400IU

### 3.3 Quality assessment of the included studies

**FIGURE 2:**
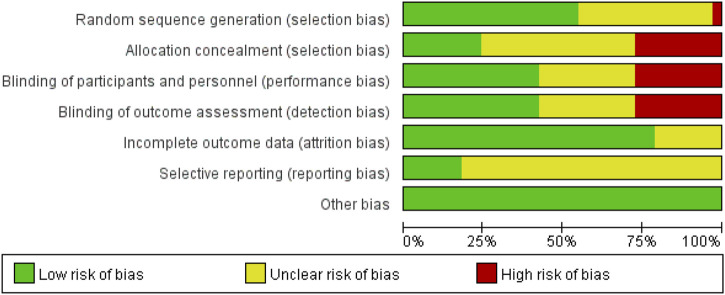
Risk of bias plot over all studies.

Although all the studies included in this meta-analysis were randomized controlled trials, some studies had a higher risk of bias due to the use of list order allocation rather than true randomization (Dundar et al., 2009). Additionally, several studies employed an open-label design without adequate use of double-blind or triple-blind methods, which posed a higher risk of bias (Cesareo et al., 2015; Frediani et al., 1998; Iwamoto and Sato, 2014; Iwamoto et al., 2003; Kim et al., 2014; Leung et al., 2005; Lyritis et al., 1997; Masud 1998; Tanakol et al., 2007). Furthermore, certain outcomes were assessed as having a medium risk of bias because the studies did not clearly describe the relevant specifics (Figure 2).

### 3.4 Bone density analysis results from different sites

**FIGURE 3:**
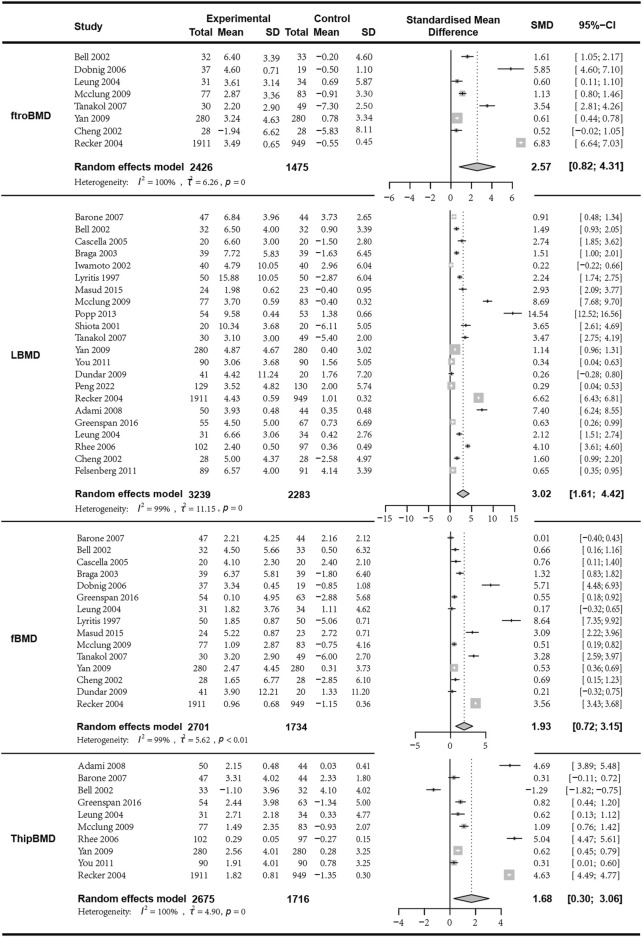
Forest plot for LBMD, ftroBMD, fBMD,ThipBMD changes.

We first analyzed the changes in LBMD before and after treatment between the combination group and the monotherapy group. A total of seven articles with 622 patients were included. Before conducting the meta-analysis, it was important to ensure that there was no difference in the baseline values between the combined treatment and the control treatment. The results indicated that the combination of bisphosphonates and VitD was superior to the monotherapy group, with a significant difference (*I* ^*2*^ = 99.40%, *p* = 0; random effects model; SMD [95% CI] = 3.02 [1.61, 4.42], *p* < 0.01, Low GRADE). Similarly, the combination of bisphosphonates and VitD shows a significant advantage over monotherapy in improving ftroBMD (*I* ^*2*^ = 99.70%, *p* = 0; random effects model; SMD [95% CI] = 2.57 [0.82, 4.31], *p* = 0.04, Low GRADE), fBMD (*I* ^*2*^ = 99.10%, *p* < 0.01; random effects model; SMD [95% CI] = 1.93 [0.72, 3.15], *p* = 0.01, Low GRADE), and ThipBMD (*I* ^*2*^ = 99.60%, *p* = 0; random effects model; SMD [95% CI] = 1.68 [0.30, 3.06], *p* = 0.02, Low GRADE) (Figure 3) (Supplementary Material S3).

### 3.5 Results of bone formation marker analysis

#### 3.5.1 Pooled analysis for the changes in osteocalcin

**FIGURE 4:**
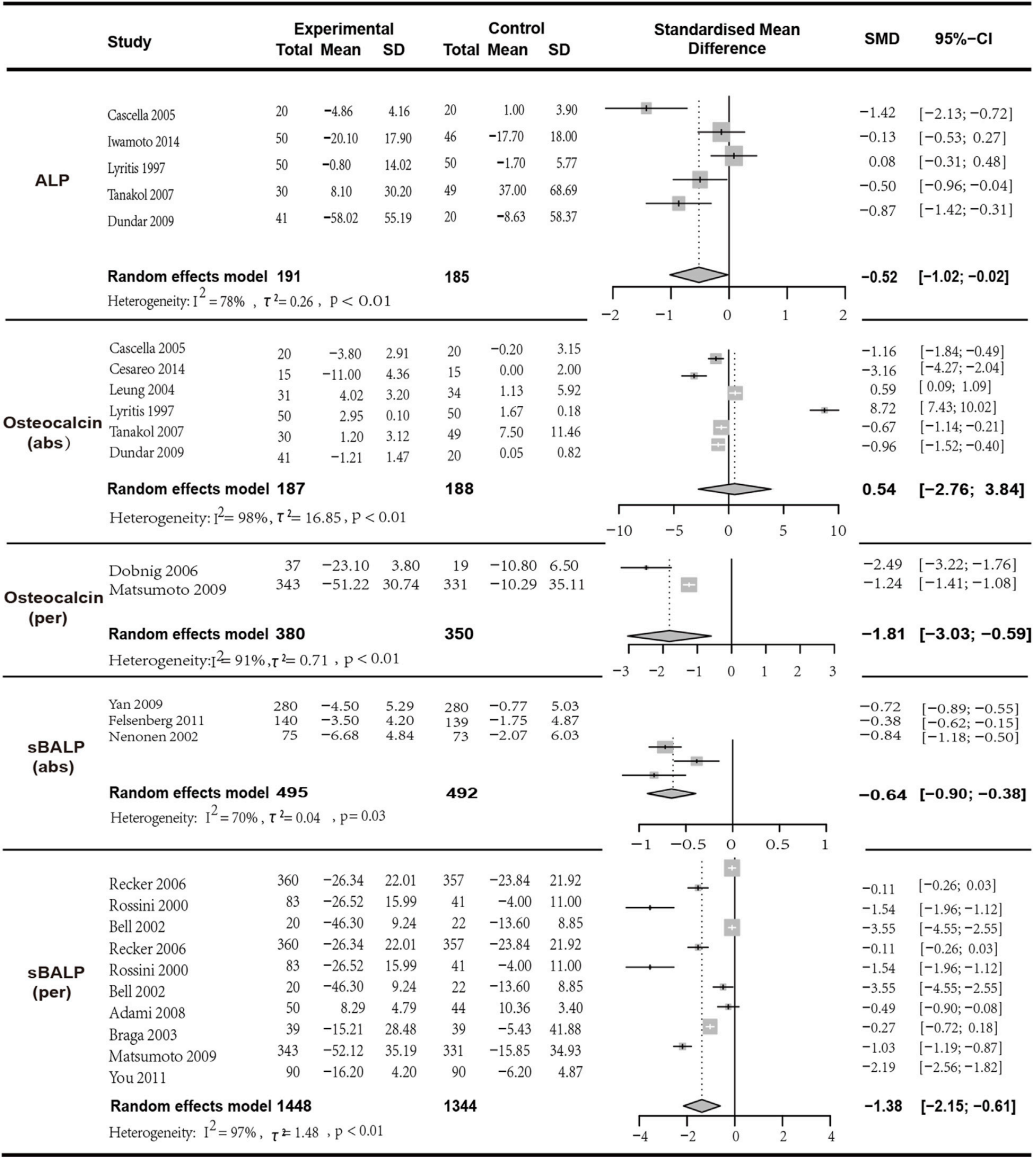
Forest plot for ALP,Osteocalcin (abs),Osteocalcin (per),sBALP (abs) and sBALP (per) changes.

Meta-analysis included eight studies, of which six studies with a total of 375 patients provided data on the changes in mean osteocalcin values and were included in the analysis (I^2^ = 97.90%, *p* < 0.01; random effects model; SMD [95% CI] = 0.54 [-2.76, 3.84], *p* = 0.75, Low GRADE). Notably, the *p*-value of the SMD indicating that the results of this analysis did not have statistical significance and should be referred to with caution. Additionally, two studies with a total of 750 patients provided data on the percentage change in osteocalcin (I^2^ = 90.70%, *p* = 0.01; random effects model; SMD [95% CI] = −1.81 [-3.03, −0.59], *p* = 0.01,Low GRADE). The results indicated that although the combination of the two drugs was superior to monotherapy in terms of average changes in osteocalcin levels, the SMD was limited. The analysis of percentage changes in osteocalcin, which included more participants, suggests that monotherapy was superior to combined therapy, indicating that the combined treatment of VitD and bisphosphonates may not bring positive effects in improving patients’ osteocalcin levels (Figure 4).

#### 3.5.2 Pooled analysis for the changes in ALP and sBALP

**FIGURE 5:**
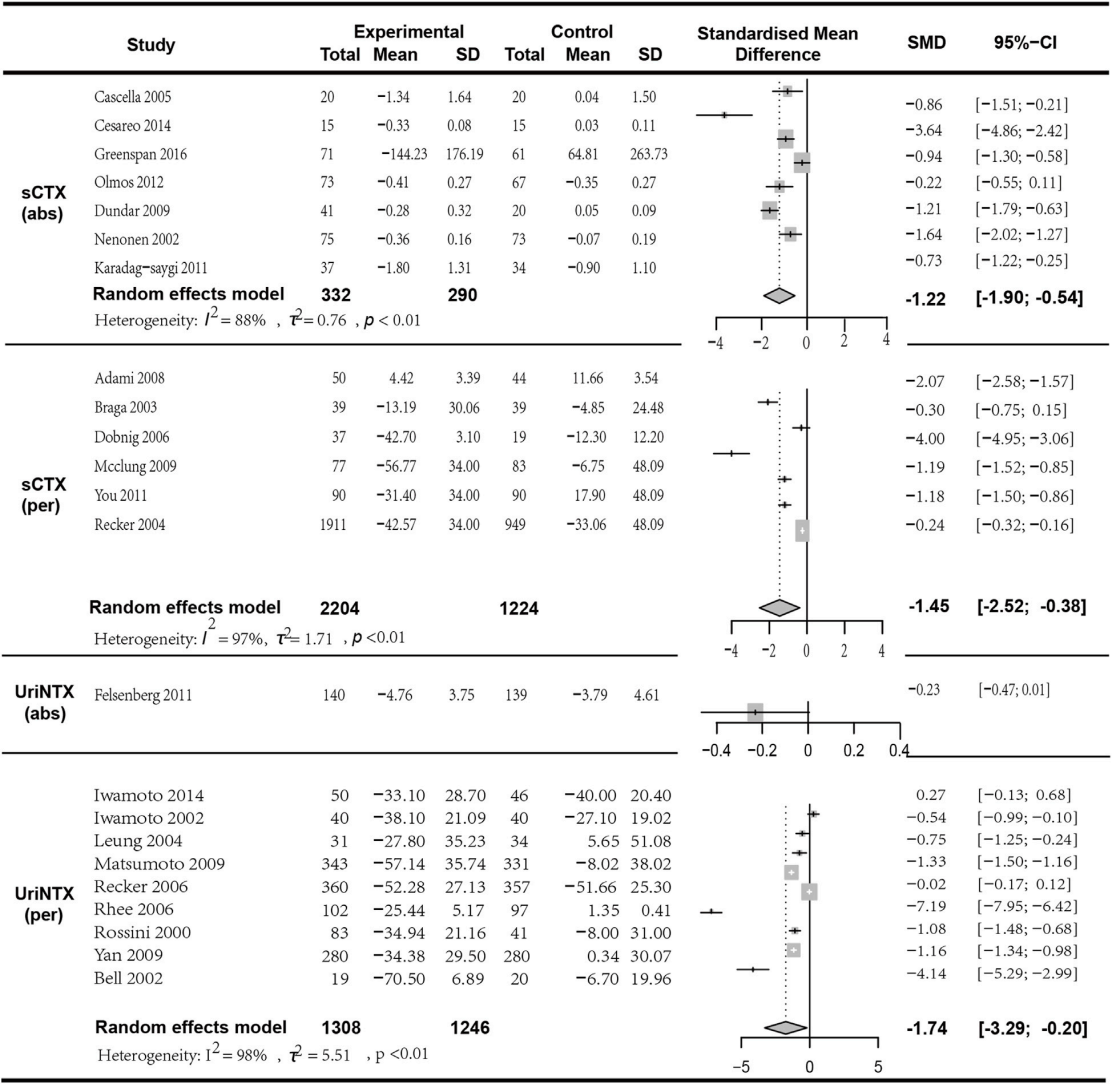
Forest plot for sCTX (abs), sCTX (per),uriNTX (abs) and uriNTX (per) changes.

In the analysis of ALP, five articles with a total of 376 patients were included. The results indicated that the combination of bisphosphonates and VitD was more effective than monotherapy with a significant difference (*I* ^*2*^ = 78.40%, *p* = 0.01; random effects model; SMD [95% CI] = −0.52 [-1.02, −0.02], *p* = 0.04; as shown in Figure 5. Low GRADE). Similarly, three studies with a total of 987 patients provided data on the changes in mean sBSAP values (*I* ^*2*^ = 70.30%, *p* = 0.03; random effects model; SMD [95% CI] = −0.64 [-0.90, −0.38], *p* < 0.01; as shown in Figure 5. Moderate GRADE). Two studies with a total of 750 patients provided data on the percentage change in sBALP (*I* ^*2*^ = 97.00%, *p* < 0.01; random effects model; SMD [95% CI] = −1.38 [−2.15, −0.61], *p* = 0.01, Very low GRADE). The results indicated that bisphosphonates combined with VitD was superior to monotherapy with a significant difference (Figure 4).

### 3.6 Results of bone resorption marker analysis

#### 3.6.1 Pooled analysis for the changes in sCTX and uriNTX

**FIGURE 6:**
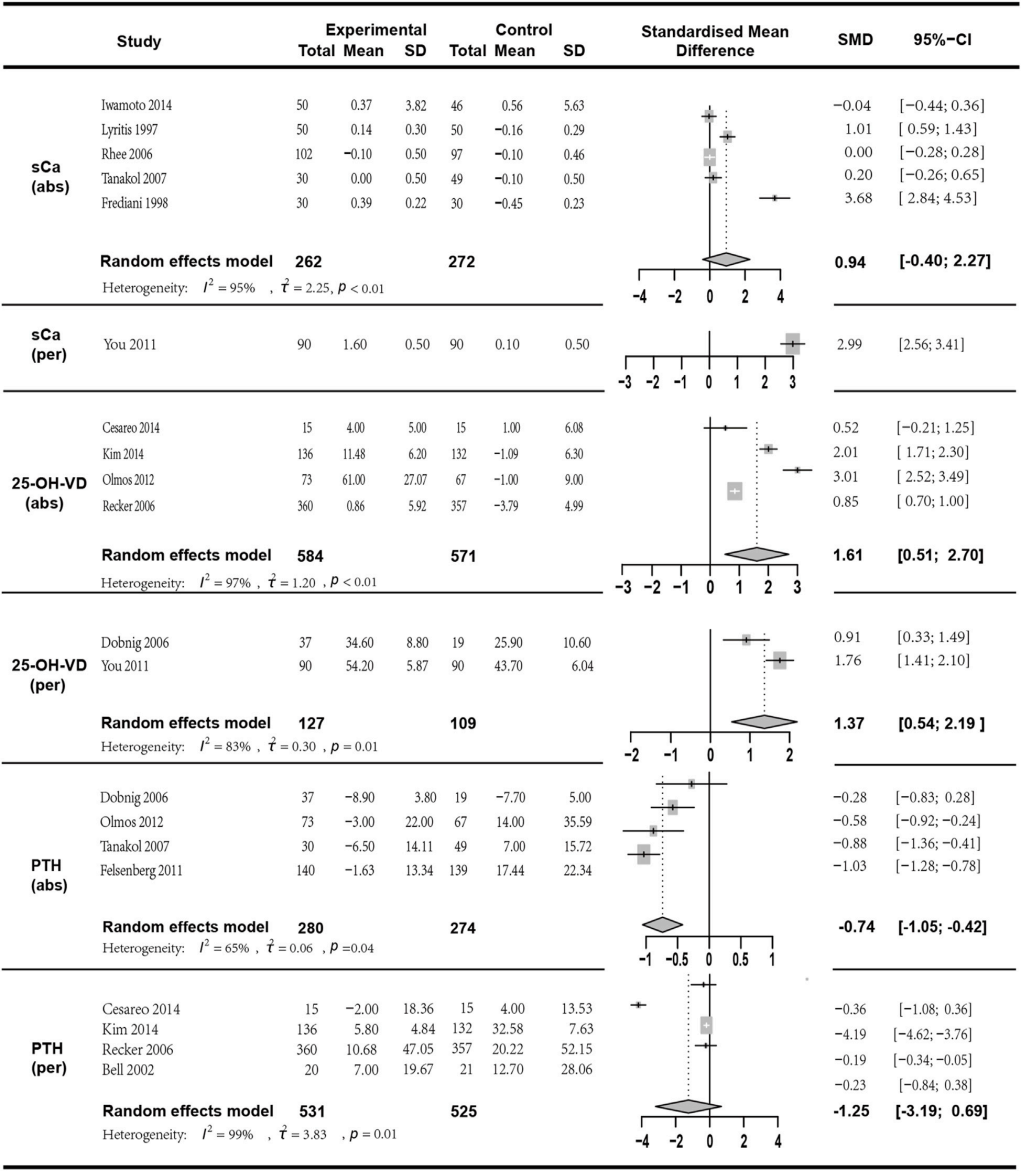
Forest plot for sCa (abs), sCa (per),25-OH-VD (abs),25-OH-VD (per),PTH(abs) and PTH(per) changes.

In the analysis of the mean change in sCTX, seven studies with a total of 622 patients were included. The results indicated that the combination of bisphosphonates and VitD was more significantly effective than monotherapy (*I* ^*2*^ = 88.50%, *p* < 0.01; random effects model; SMD [95% CI] = −1.22 [−1.90, −0.54], *p* = 0.01; as shown in Figure 6. Low GRADE). In the analysis of the percentage change in sCTX, six articles with a total of 3428 patients were included. The results indicated that the combination of bisphosphonates and VitD was superior to monotherapy with a significant difference (I^2^ = 88.50%, *p* < 0.01; random effects model; SMD [95% CI] = −1.22 [−1.90, −0.54], *p* = 0.0; as shown in Figure 5.Low GRADE). Similarly, in the analysis of the mean change in UriNTX (random effects model; SMD [95% CI] = −0.23 [−0.47, 0.01], *p* = 0.06, High GRADE) and the percentage change in UriNTX (*I* ^*2*^ = 98.40%, *p* < 0.01; random effects model; SMD [95% CI] = −1.74 [−3.29, 0.20], *p* = 0.03, Low GRADE), we also found that combination of bisphosphonates and VitD has an advantage over monotherapy. However, there was no statistical difference in the mean change in UriNTX (*p* > 0.05) (Figure 5).

### 3.7 Results of bone calcium metabolism marker analysis

We conducted a meta-analysis of sCa (six studies), 25-OH-VD (six studies), and PTH (eight studies). The results are as follows:

The sCa mean change was reported in five studies with a total of 534 participants (*I* ^*2*^ = 94.90%, *p* < 0.01; random effects model; SMD [95% CI] = 0.94 [−0.40; 2.27], *p* = 0.17, Low GRADE). Since the *p*-value of the SMD was greater than 0.05, indicating no statistical significance, this result should be interpreted with caution. sCa percentage change was reported in one study with a total of 180 participants (SMD [95% CI] = 2.99 [2.56, 3.41], *p* < 0.01, High GRADE).

25-OH-VD mean change was analyzed in four studies with a total of 1155 participants (*I* ^*2*^ = 97.10%, *p* < 0.01; random effects model; SMD [95% CI] = 1.61 [0.51, 2.70], *p* = 0.01, Low GRADE). 25-OH-VD percentage change was reported in two studies with a total of 236 participants (*I* ^*2*^ = 83.50%, *p* = 0.01; random effects model; SMD [95% CI] = 1.37 [0.54, 2.19], *p* = 0.01, Low GRADE).

PTH mean change was reported in four studies with a total of 554 participants (*I* ^*2*^ = 64.70%, *p* = 0.04; random effects model; SMD [95% CI] = −0.74 [−1.05, −0.42], *p* < 0.01, Moderate GRADE). PTH percentage change was discussed in four studies with a total of 1056 participants. (*I* ^*2*^ = 99.00%, *p* < 0.01; random effects model; SMD [95% CI] = −1.25 [−3.19, 0.69], *p* = 0.21, Low GRADE). The results indicated (Figure 6) that these three indicators in the combined therapy group were superior to those in the monotherapy group, and except for the percentage change in PTH (*p* > 0.05), all had statistical significance.

### 3.8 Safety

**FIGURE 7:**
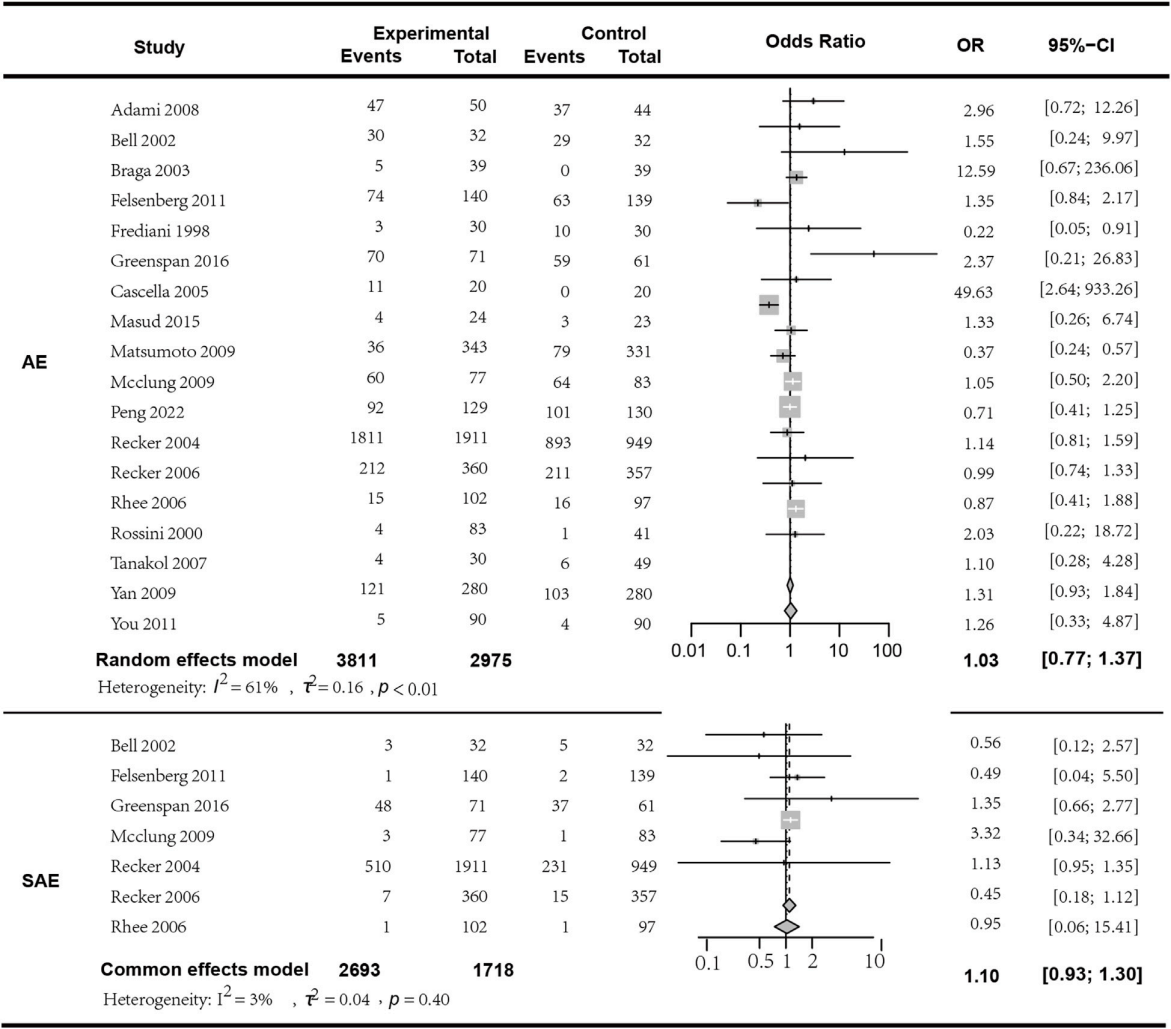
Forest plot for AE and SAE with combination treatment *versus* monotherapy.

In the analysis of adverse events (AEs), a total of 18 studies involving 6606 patients were included. The results indicated that the occurrence of AEs with bisphosphonate combination therapy in conjunction with Vitamin D does not statistically significantly differ from monotherapy (*I* ^*2*^ = 61.00%, *p* < 0.01; random effects model; OR [95% CI] = 1.03 [0.77, 1.37], *p* = 0.85, see Figure 7. Moderate GRADE). In the analysis of serious adverse events (SAEs), seven studies encompassing 4411 patients were included. The findings showed no statistically significant difference in the occurrence of SAEs between bisphosphonate plus Vitamin D therapy and monotherapy (*I* ^*2*^ = 3.00%, *p* = 0.40; fixed effects model; OR [95% CI] = 1.10 [0.93, 1.30], *p* = 0.28; Figure 7. High GRADE). To clarify the specific differences between combination therapy and each individual component of Vitamin D or bisphosphonates, we conducted a subgroup analysis with different monotherapy comparisons within the control group, and found no significant difference in the subgroup analysis regarding both adverse reactions and serious adverse reactions (*p* > 0.05, detailed in the attachment). The most commonly reported adverse reactions were: 1) acute-phase reactions following drug infusion: local pain, fever; 2) gastrointestinal adverse events; 3) influenza-like symptoms, etc. We observed that the rate of adverse events might increase with higher doses of bisphosphonates in combination therapy. For instance, in the study by Adami et al. (to be cited), the incidence rate of moderate to severe local pain was lower in the placebo group (27.8%) compared to the three active groups: 50 mg group 77.8%, 25 mg group 71.9%, and 12.5 mg group 57.4%. Therefore, a more comprehensive safety assessment may be necessary when using relatively higher doses of bisphosphonate therapy. Detailed in the Supplementary Table S1 Specific adverse effects of the included studies.

### 3.9 Subgroup analysis

Additionally, to clarify the specific differences between combined treatment and VitD or bisphosphonates, we conducted subgroup analyses of different monotherapy indicators in the control group. We found that there were no significant differences between the bisphosphonate monotherapy group and the VitD monotherapy group in subgroup analyses of LBMD, fBMD, ThipBMD, ALP, PTH(abs), PTH(per), 25-OH-VD (abs), and sCa (abs) (*p* > 0.05). However, in the subgroup analyses of sBALP (per), sCTX (abs), and Uri (per), the group treated with bisphosphonate monotherapy showed significant differences compared to the group treated with vitD monotherapy (*p* < 0.05). Detailed results of the subgroup analyses are shown in the attachment.

### 3.10 Sensitivity analysis

To test the robustness of the results, we performed sensitivity analyses on the above indicators. In this meta-analysis, each sensitivity analysis showed that the results did not change with the exclusion of any single study (attachment).

### 3.11 Publication bias

For the meta-analyses of LBMD, fBMD, ThipBMD, and sBALP, which all included 10 or more studies, we performed Egger’s test for publication bias. The results showed that significant publication bias was detected for sBALP (Egger *p* = 0.02), but not for the other indicators (Egger *p* > 0.05). Therefore, we applied the trim-and-fill method to correct the results for sBALP. The corrected SMD [95% CI] was −0.50 [−1.49; 0.49], *p* = 0.32, which differed significantly from the unadjusted result. This suggests that the unadjusted result was not reliable due to the presence of publication bias. The meta-analysis results for this outcome measure need to be handled with great caution (attachment).

## 4 Discussion

VitD plays a crucial role in regulating the homeostasis of calcium and phosphorus metabolism and optimizing osteoblast activity. In mature osteoblasts, the 1,25(OH)_2_D3 signaling pathway can promote osteoblast differentiation and the expression of osteocalcin (OCN), thereby facilitating bone mineralization (Morris et al., 2012). It also decreases the expression of the receptor activator of nuclear factor-κB ligand (RANKL), which is a key factor in osteoclast differentiation, and enhances the expression of its antagonist, osteoprotegerin (OPG), thereby indirectly inhibiting bone resorption (Horwood et al., 1998). Additionally, VitD can enhance bone mineralization by promoting the maturation of osteoblasts, regulating their activity, and inducing the expression of bone formation-related genes (CYP-24) (van Driel et al., 2006). Bisphosphonates, as inhibitors of bone resorption, have a structure where the R1 group determines the rapid and selective binding of bisphosphonates to the surface of bone minerals, while the R2 group plays a decisive role in countering bone resorption (Diel et al., 1998). The R2 group can directly act on osteoblasts, promoting osteoblast differentiation through the JNK (N-terminal kinases) and ERK (Extracellular signal-regulated kinases) signaling pathways (Fu et al., 2008). It also secretes osteoclast inhibitory factors to inhibit the activity of osteoclasts (Rogers et al., 2020). As a result, bone metabolic markers such as β-isomerized C-terminal telopeptides (β-CTX), procollagen type I N-propeptide (PINP), Human N-terminal middle osteocalcin (N-MID-OT) are reduced (Bell et al., 2016; Tan et al., 2016; Lipton et al., 2016; Nishimukai et al., 2017). Furthermore, nitrogen-containing bisphosphonates can inhibit the enzyme systems in the Mevalonate pathway, suppress the biosynthesis of isoprenoids, and induce osteoclast apoptosis (Compston, 1994). Therefore, VitD and bisphosphonates can act synergistically through different signaling pathways to regulate calcium-phosphorus homeostasis and exert antifracture effects. Additionally, in endocrine pathways, high concentrations of PTH can reduce the anti-resorptive effects of bisphosphonates, while VitD coordinates with fibroblast growth factor 23 (FGF23) produced by PTH, resulting in a balance between them. Consequently, the combination of VitD and bisphosphonate therapy can significantly enhance the efficacy against osteoporosis (Mosali et al., 2014).

The results of this study indicate that there were no significant statistical differences in the mean changes of Osteocalcin, UriNTX, and sCa indicators, as well as the percentage change of PTH (*p* > 005). This result may be due to the insufficient sample size of the studies included. However, changes in other indicators before and after treatment were statistically significant. In terms of improving bone density, the combination therapy group of VitD and bisphosphonates showed a more significant advantage over the monotherapy groups, particularly in LBMD and froBMD. In terms of regulating serum biomarker concentrations, the combination group showed more pronounced effects on increasing levels of ALP, 25-OH-VD, and sCa, as well as on reducing levels of sBALP, sCTX, and UriNTX. Although there were differences in the SMDs between the average change group and the percentage change group for sCa, sBALP, and UriNTX, with the percentage change group showing more prominent effects, this phenomenon may be related to the different forms of representation for the chosen endpoint variables. It is noteworthy that the effects of VitD combined with bisphosphonates on increasing Osteocalcin levels were not superior to those of monotherapy groups, solely based on the average value changes. Additionally, the percentage change of Osteocalcin also failed to demonstrate statistical significance, suggesting that there may be no additional advantages of combined therapy in improving Osteocalcin levels. Considering the above results, the combination of VitD and bisphosphonates appears to be more effective than monotherapy in the overall treatment of osteoporosis. The potential mechanisms may include the modulation of calcium-phosphorus homeostasis by adequate VitD, which participates in the regulation of bone metabolism and maximizes the therapeutic effects of bisphosphonates through different signaling pathways (Nishimukai et al., 2017; Compston, 1994; Mosali et al., 2014). Furthermore, secondary hyperparathyroidism induced by VitD deficiency and the accompanying increase in PTH levels can diminish the efficacy of bisphosphonates. The combined therapy may optimize treatment outcomes indirectly by regulating PTH levels (Mosali et al., 2014).

Research by Andrew C Karaplis and others found that the most common drug-related adverse events during the combination therapy of vitamin D3 and alendronate for osteoporosis were gastrointestinal disorders (Karaplis et al., 2011). Thawee Songpatanasilp and others reported that adverse reactions such as dyspepsia, myalgia, and headache were associated with the combined use of alendronate and vitamin D3 (Songpatanasilp et al., 2018). The study by You and others found no significant difference in the incidence of adverse reactions between the combination therapy of alendronate and alfacalcidol for osteoporosis and the monotherapy with alfacalcidol alone (You et al., 2011). Kim and others also supported this view, with no statistically significant difference in the incidence of drug-related adverse events between combined therapy and monotherapy (Kim et al., 2014). Additionally, studies have suggested that reducing the dose or frequency of alendronate during combination therapy can reduce the occurrence of symptoms (Bone et al., 2000; Schnitzer et al., 2000). From a mechanism of action perspective, bisphosphonates do have some factors that may contribute to the occurrence of adverse events. Nitrogen-containing bisphosphonates like alendronate and zoledronate inhibit key enzymes in the mevalonate pathway, such as farnesyl pyrophosphate synthase (FPP), leading to the accumulation of isopentenyl pyrophosphate (IPP) within cells. This blocks the isoprenylation of small GTPases, thereby affecting various cellular functions. The accumulated IPP in monocytes and macrophages activates and proliferates γδ T cells, causing the release of pro-inflammatory cytokines TNFα and IL6, which can lead to an acute systemic inflammatory response, manifested as headaches, myalgia, flu-like symptoms (Roelofs et al., 2006). Additionally, the gastrointestinal tract is a major repository for γδ T cells (Hewitt et al., 2005), which may explain the gastrointestinal disorders like dyspepsia associated with oral bisphosphonate use. A study by R E Hewitt and others found that pretreatment with antipyretics or histamine receptor antagonists can reduce the incidence of these symptoms (Hewitt et al., 2005).

Our meta-analysis is the first to systematically evaluate and quantitatively analyze the effects of combined treatment with VitD and bisphosphonates on postmenopausal women with osteoporosis. The number of studies included is abundant, and the participants are well-matched, which enhances the comparability of the results. We have separately merged studies reporting numerical changes and percentage changes for the same endpoint measures, improving the comprehensiveness of the data analysis. This study also has certain limitations: although the results are robust after sensitivity analysis, there is considerable heterogeneity among the included studies, which may be due to differences in baseline characteristics of the patients included in each study, variations in regions, different drug dosages and frequencies, varying study observation periods, different ages of patients, and inconsistent efficacy of the same treatment regimen, leading to different amounts of change in endpoint measures and increased heterogeneity in results. In conducting this study, we acknowledge that the scope of the literature and data included is finite, which may limit our in-depth analysis of the details of medication use. The inherent limitations of the data make it challenging to explore all sources of potential heterogeneity exhaustively. The sources of heterogeneity in the analysis may be multifaceted, such as differences in the comorbidities across different populations, basic medications or supplements, the types and manufacturing of vitamin D and bisphosphonates of interest, and the methods for measuring results by different institutions. Although we ensured that the PICOS background of different groups in the same study was consistent during literature screening, to ensure that the differences between groups can be fully explained by the intervention of interest, the above-mentioned differences between studies may still lead to unknown biases. Although our analysis results were proven to be robust by subgroup analysis and sensitivity analysis, necessary caution is still needed. Furthermore, our literature search was confined to a few databases in both Chinese and English, which may lead to the omission of certain studies and introduce the potential for publication bias. Whether used alone or in combination, different treatment courses or drug doses can lead to different risks of adverse events, highlighting the urgency of finding the optimal dosage, frequency, and course of medication. Additionally, older individuals often have multiple coexisting diseases (Hu et al., 2015), so attention must be paid to interactions between different types of drugs during use. In recent years, with the rapid development of molecular biology, immunology, pharmacology, and other fields, there has been significant progress in understanding the pathogenesis of osteoporosis. The development of new drugs and the use of different drugs in combination have provided clinicians with diverse treatment options for osteoporosis.

## 5 Conclusion

Our analysis suggests that the combination therapy of VitD and bisphosphonates is more effective than monotherapy for treating osteoporosis in postmenopausal women, providing a scientific basis for the better treatment of osteoporosis. However, due to the limitations of this study, further and more in-depth research on this topic is needed in the future.

## Data Availability

The original contributions presented in the study are included in the article/Supplementary Material, further inquiries can be directed to the corresponding author.
