# Supplementary material for: Efficacy of combination therapy of vitamin D and bisphosphonates in the treatment of postmenopausal osteoporosis: a systematic review and meta-analysis
Source: Front Pharmacol. 2024 Nov 21;15:1422062. doi: 10.3389/fphar.2024.1422062 (PMC11617160; doi:10.3389/fphar.2024.1422062)
Supplement: Supplementary file 1 [file DataSheet1.zip › Table S2.DOCX]

**Table S2** Drug regimens of included studies.

| **Study** | **Basic treatment** | **Monotherapy** | **Combination therapy** | **Duration** |
| --- | --- | --- | --- | --- |
| Adami et al. | Ca 1g/D | VD 800IU/D | Neri 12.5 or 25 or 50mg/M and VD 800IU/D | 12 Months |
| Barone et al. | Ca 1g/D | Alen 10mg/D | Alen 10mg/D and Cal 0.5μg/D | 12 Months |
| Bell et al. | Ca 0.5g/D | VD 500IU/D | Alen 10mg/D and VD 500IU/D | 24 Months |
| Braga et al. | Ca 0.5g/D | VD 400IU/D | Neri 50mg/M and VD 400IU/D | 24 Months |
| Cascella et al. | Ca 0.5g/D | VD 400IU/D | Neri 25mg/M and VD 400IU/D | 24 Months |
| Cesareo et al. | Ca 1g/D | VD3 400 IU/D | Alen 10mg/D and VD3 400 IU/D | 24 Months |
| Cheng et al. | Ca 0.6g/D | VD 1000IU/D | Alen 10mg/D and VD 1000IU/D | 6 Months |
| Dobnig et al. | Ca 1.0-1.2g/D | VD 400-800IU/D | Rise 5mg/D or Alen 10mg/D and VD 400-800IU/D | 12 Months |
| Dundar et al. | Ca 1g/D | VD 400IU/D | Rise 5mg/D and VD 400IU/D | 12 Months |
| Felsenberg et al. | Ca 0.5g/D | Alen 10mg/D | Alen 10mg/D and Alfa 1μg/D | 36 Months |
| Frediani et al. | Ca 0.5g/D | Alen 10mg/D or cal 0.5μg/D | Alen 10mg/D and cal 0.5μg/D | 24 Months |
| Greenspan et al. | Ca 1.2g/D | VD 800IU/D | Zole 5mg/D and VD 800IU/D | 24 Months |
| Iwamoto et al. | NR | Alen 5mg/D or Rise 2.5mg/D | Alen 5mg/D or Rise 2.5mg/D and VD 0.75μg/D | 6 Months |
| Iwamoto et al. | Ca 0.8g/D | Etid 2800 mg/3M | Etid 2800 mg/3M and Alfa 1μg/D | 12 Months |
| Karadag-saygi et al. | Ca 0.6g/D | VD 400IU/D | Rise 35mg/W and VD 400IU/D | 6 Months |
| Kim et al. | Ca 0.2g/D | VD 800IU/D | Alen 10mg/D and VD 800IU/D | 16 Months |
| Leung et al. | Ca 0.5g/D | VD 400IU/D | Rise 5mg/D and VD 400IU/D | 12 Months |
| Lyritis et al. | Ca 0.5g/D | Cal 0.4μg/D | Etid 400mg/D and cal 0.4μg/D | 48 Months |
| Masud et al. | Ca 0.5g/D | Etid 0.4g/D | Etid 0.4g/D and Cal 0.5 µg/D | 12 Months |
| Matsumoto et al. | Ca 0.6g/D | VD 200IU/D | Mino 1mg/D and VD 200IU/D | 24 Months |
| Mcclung et al. | Ca 0.5g/D | VD 400IU/D | Iban 5mg/D and VD 400IU/D | 12 Months |
| Nenonen et al. | Ca 0.63g/D | VD 200IU/D | Alen 5mg/D and VD 200IU/D | 12 Months |
| Olmos et al. | NR | Alen 70mg/W | Etid 0.4g/D and Cal 0.3mg/W | 3 Months |
| Peng et al. | Ca 0.5g/D | VD3 200IU/D | Mino 1mg/D and VD3 200IU/D | 12 Months |
| Popp et al. | Ca | VD3 | Zole 5mg/Y and VD3 | 36 Months |
| Recker et al. | Ca 0.5-0.6g/D | Alen 10mg/D | Alen 10mg/D and VD3 400IU/D | 15 Months |
| Recker et al. | Ca 0.5g/D | VD 400IU/D | Iban 1 or 0.5mg/3M and VD 400IU/D | 36 Months |
| Rhee et al. | NR | Alfa 1µg/D | Alen 5mg and Cal 0.5µg/D | 24 Months |
| Rossini et al. | Ca 0.5g/D | VD 440IU/D | Alen 20mg/W or Alen 10mg/D and VD 440IU/D | 12 Months |
| Shiota et al. | Ca 2g/D | Alfa 0.5μg/D | Etid 200mg/D and Alfa 0.5μg/D | 24 Months |
| Tanakol et al. | Ca 0.5g/D | VD 400IU/D | Clod 400mg/D and VD 400IU/D | 36 Months |
| Yan et al. | Ca 0.5g/D | VD 200IU/D | Alen 70mg/W and VD 200IU/D | 12 Months |
| You et al. | Ca 0.6g/D and VD3 400IU | Alfa 0.5μg/D | Alen 70mg/2W and Alfa 0.5μg/D | 12 Months |
| Zole: zoledronate; Iban: ibandronate ;Rise: risedronate; Neri: neridronate; Etid: etidronate; Mino: minodronate; Alfa: alfacalcidol; Cal: calcitriol; VD:Vitamin D; Clod: Clodronic acid; Alen: alendronate | | | | |
