# Supplementary material for: Efficacy of combination therapy of vitamin D and bisphosphonates in the treatment of postmenopausal osteoporosis: a systematic review and meta-analysis
Source: Front Pharmacol. 2024 Nov 21;15:1422062. doi: 10.3389/fphar.2024.1422062 (PMC11617160; doi:10.3389/fphar.2024.1422062)
Supplement: Supplementary file 1 [file DataSheet1.zip › Table S1.DOCX]

**Table S1** Specific adverse effects of the included studies.

| **Study** | **Safety profile summary** |
| --- | --- |
| Adami et al. | The incidence of moderate to severe local pain: the placebo group (27.8%) was lower than three active groups: 77.8% in the 50 mg group, 71.9% in the 25 mg group, and 57.4% in the 12.5 mg group. |
| Barone et al. | NR |
| Bell et al. | The incidence of gastrointestinal adverse experiences and clinical fractures was not significantly different between the two groups. |
| Braga et al. | Only 13% of patients had clinical signs of an acute phase reaction (muscle pain, fever up to 37.7°C) after the first infusion of neridronate. |
| Cascella et al. | 11 patients reported flu-like symptoms in neridronate + VitD group, no adverse events were reported in the control group. |
| Cesareo et al. | NR |
| Cheng et al. | NR |
| Dobnig et al. | NR |
| Dundar et al. | NR |
| Felsenberg et al. | AEs: 1149 in the Alfa/ALN group and 1084 in the PLC/ALN group; SAEs:91 in the Alfa/ALN group and 114 in the PLC/ALN group. . |
| Frediani et al. | There were 8 cases of gastric painin in calcium group, 3 cases in alendronate + calcitriol, and 2 cases in alendronate group. |
| Greenspan et al. | There were no significant differences in number of deaths, fractures, or cardiac disorders, including atrial fibrillation. |
| Iwamoto et al. | NR |
| Iwamoto et al. | NR |
| Karadag-saygi et al. | NR |
| Kim et al. | NR |
| Leung et al. | NR |
| Lyritis et al. | NR |
| Masud et al. | 4 AEs reported in etidronate+calcitriol group,3 AEs reported in calcitriol group. |
| Matsumoto et al. | Adverse events The overall incidence of AEs was similar in both groups, as was the incidence of gastrointestinal AEs, drug-related AEs, and serious AEs. |
| Mcclung et al. | Incidence of AEs was similar between the ibandronate and placebo groups (78% and 77%).Four participants experienced serious AEs: one in the control group and three in the ibandronate group |
| Nenonen et al. | NR |
| Olmos et al. | NR |
| Peng et al. | Incidence of adverse events trial group and control group were 71.5%, 78.0%, the incidence of adverse reactions to experimental group and control group were 23.8%, 28.8%. |
| Popp et al. | NR |
| Recker et al. | 22 patients experienced one or more serious clinical adverse events; 7 (1.9%) in the ALN + VD group and 15 (4.2%) in the ALN group. |
| Recker et al. | A total of 253 patients discontinued treatment due to an adverse event: 105 from the 1 mg group, 77 from the 0.5 mg group and 71 from the placebo group. |
| Rhee et al. | 15 AEs reported in alendronate+calcitriol group,16 AEs reported in alfacalcidol group. |
| Rossini et al. | 3 patients on cyclical alendronate, 1 patient of the weekly alendronate group, and 1 patient of the control group, treatment was discontinued due to the appearance of gastrointestinal disturbances, |
| Shiota et al. | NR |
| Tanakol et al. | 8 AEs reported inClodronic acid+VD group,6 AEs reported in VD group. |
| Yan et al. | Safety The overall incidence of any adverse experiences was not significantly different between the alendronate and placebo treatment groups (43.21% vs. 36.79%) |
| You et al. | Five (5.6%) of the 90 patients in alendronate+alfacalcidol group and four (4.4%) of the 90 in alfacalcidol group reported drug-related clinical adverse events |
