# Supplementary material for: Efficacy of combination therapy of vitamin D and bisphosphonates in the treatment of postmenopausal osteoporosis: a systematic review and meta-analysis
Source: Front Pharmacol. 2024 Nov 21;15:1422062. doi: 10.3389/fphar.2024.1422062 (PMC11617160; doi:10.3389/fphar.2024.1422062)

Figure S1 Risk of bias summary in this meta-analysis.


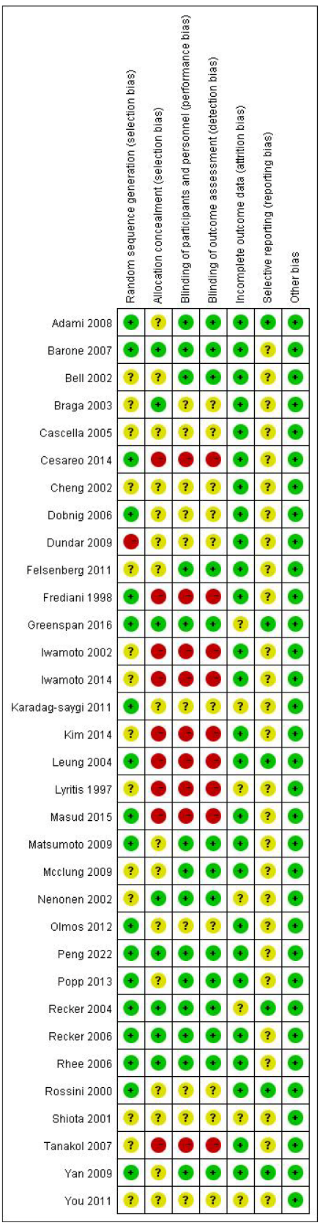


Figure S2 Subgroup analysis for LBMD


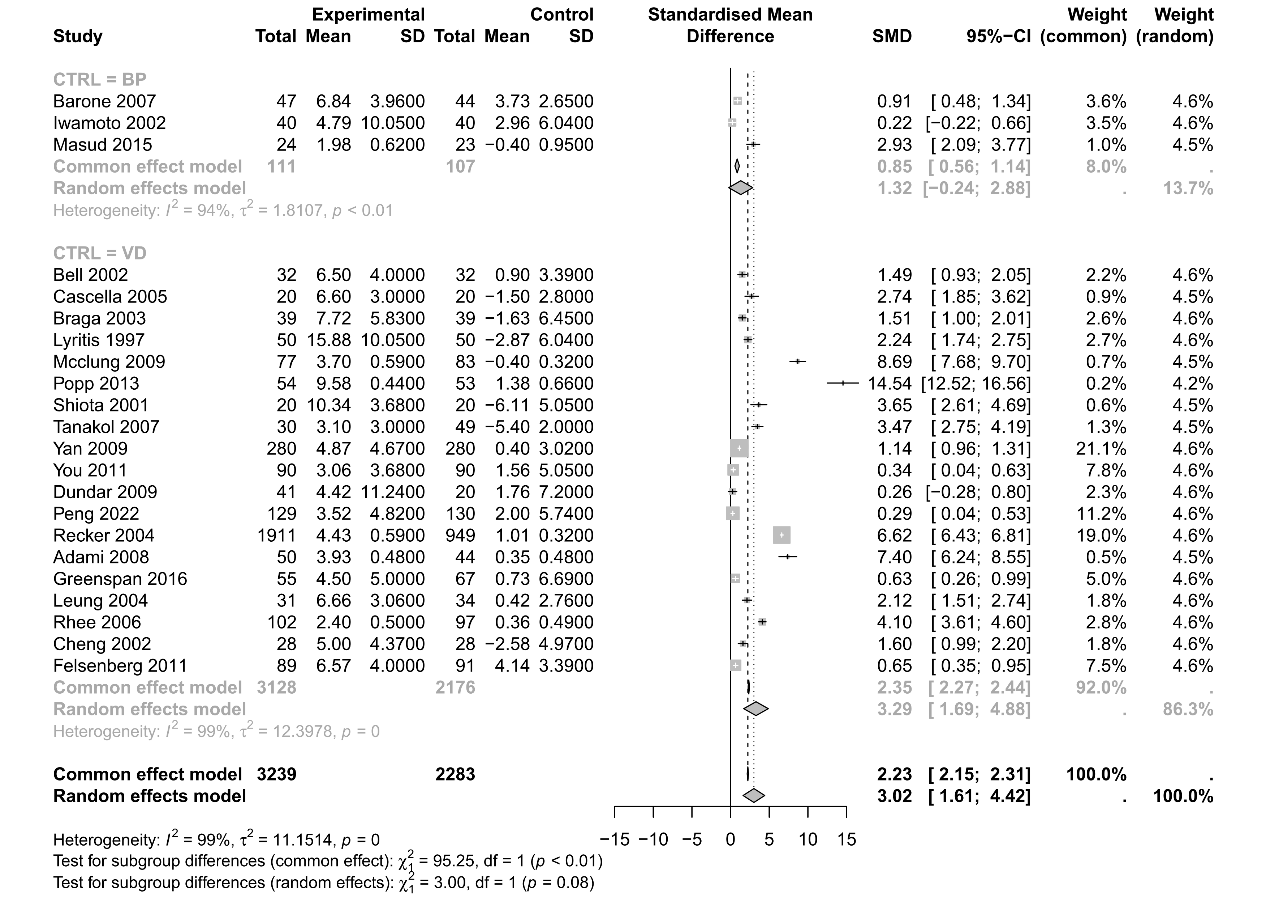


Figure S3 Subgroup analysis for fBMD


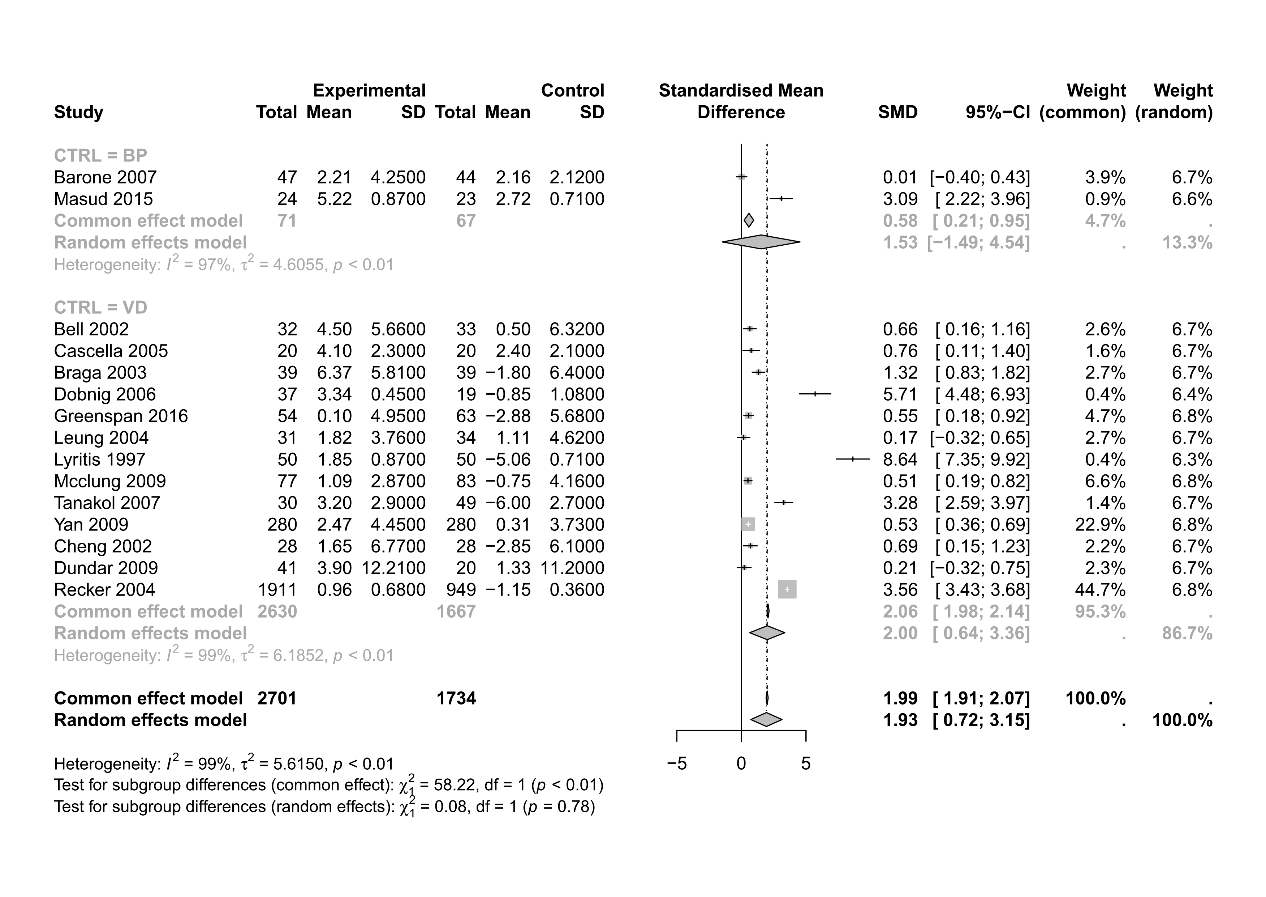


Figure S4 Subgroup analysis for ThipBMD


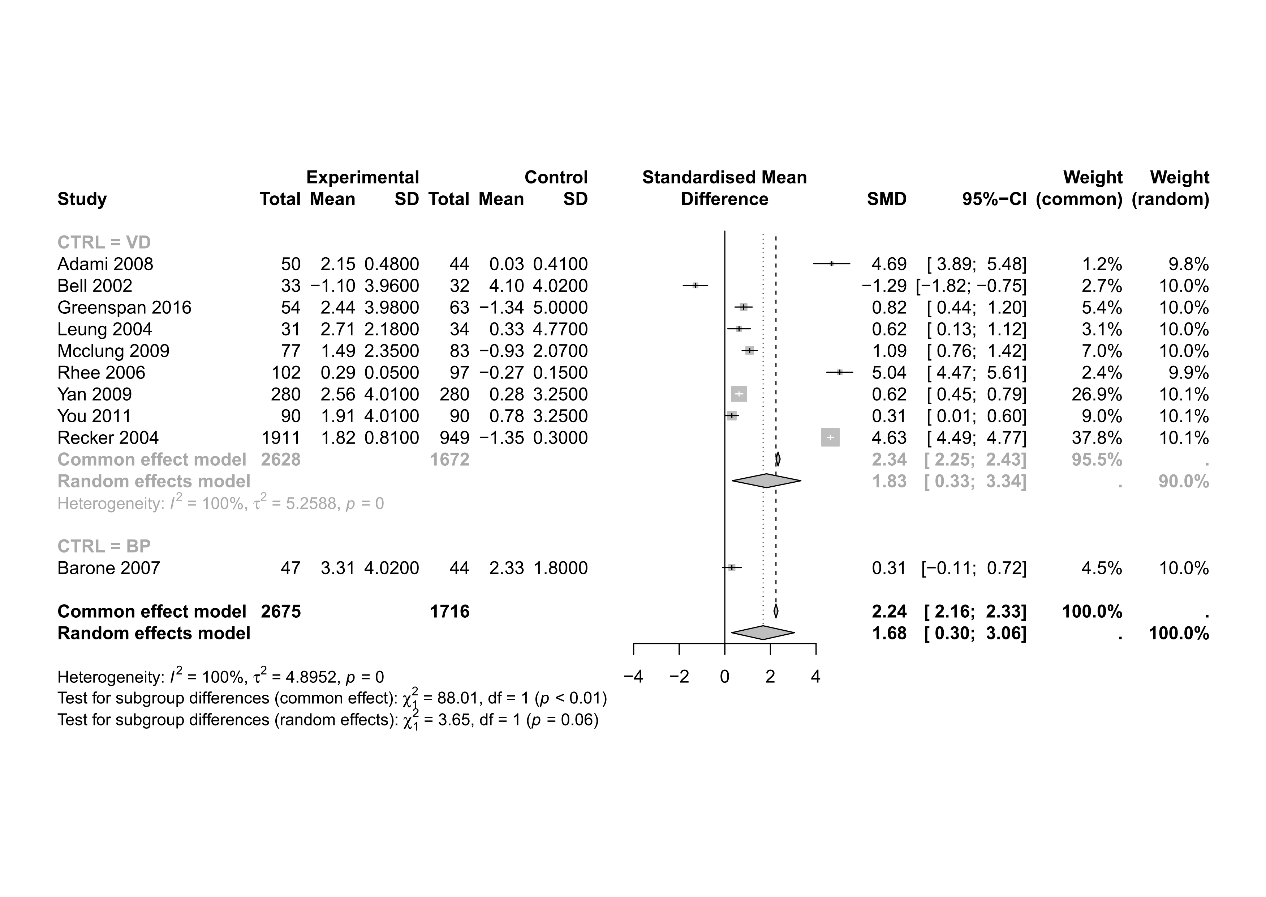


Figure S5 Subgroup analysis for ALP


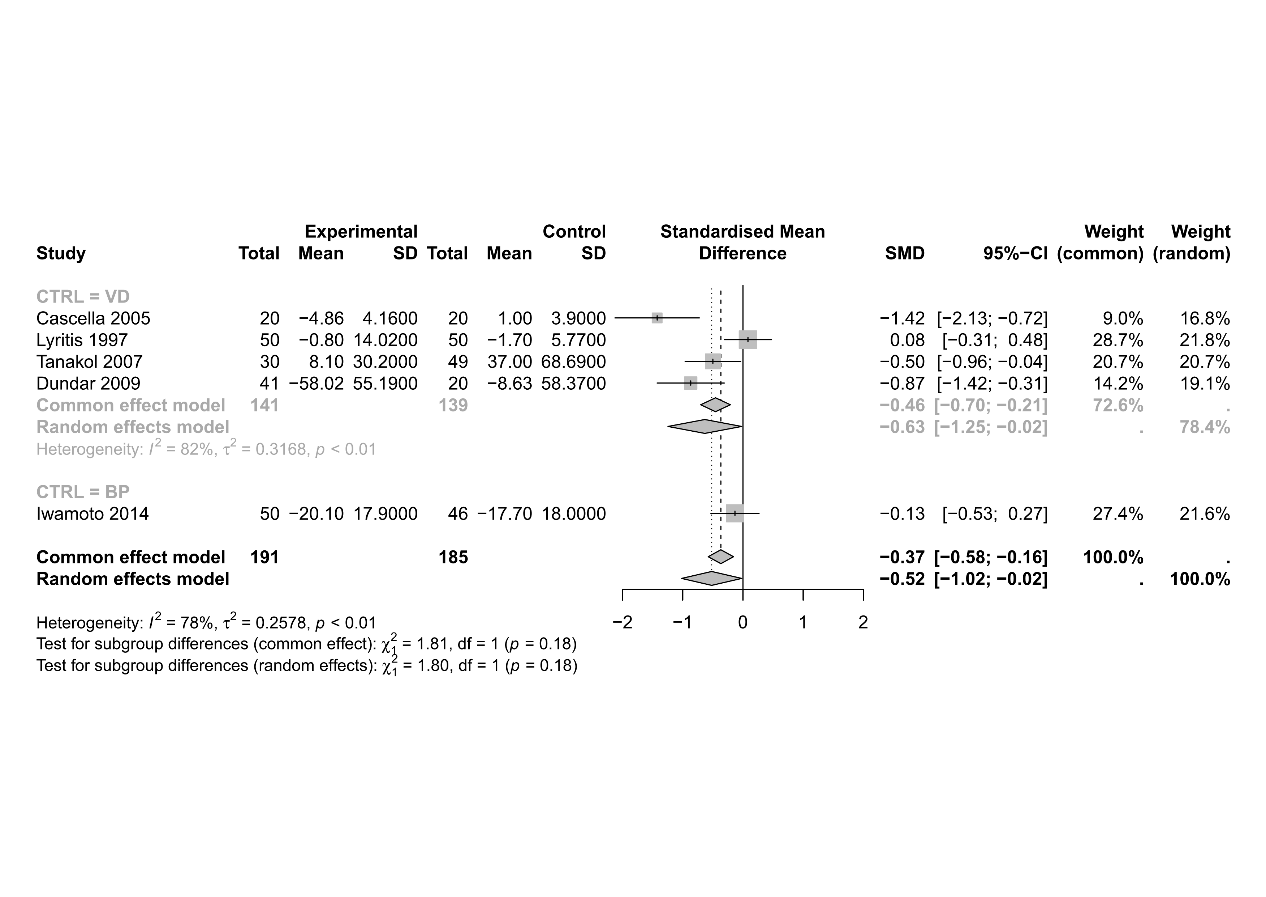


Figure S6 Subgroup analysis for25-OH-VD(abs)


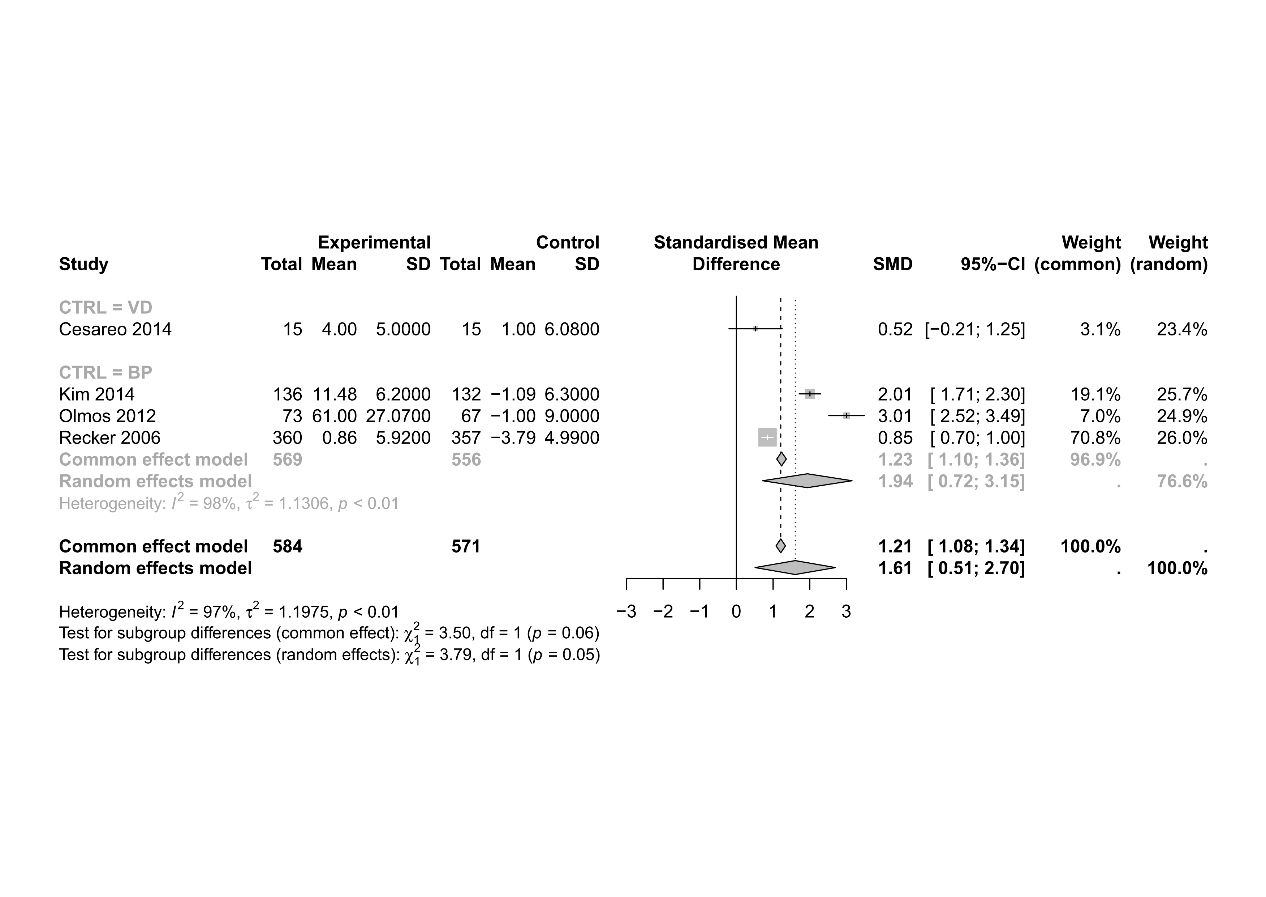
Figure S7 Subgroup analysis for PTH(abs)


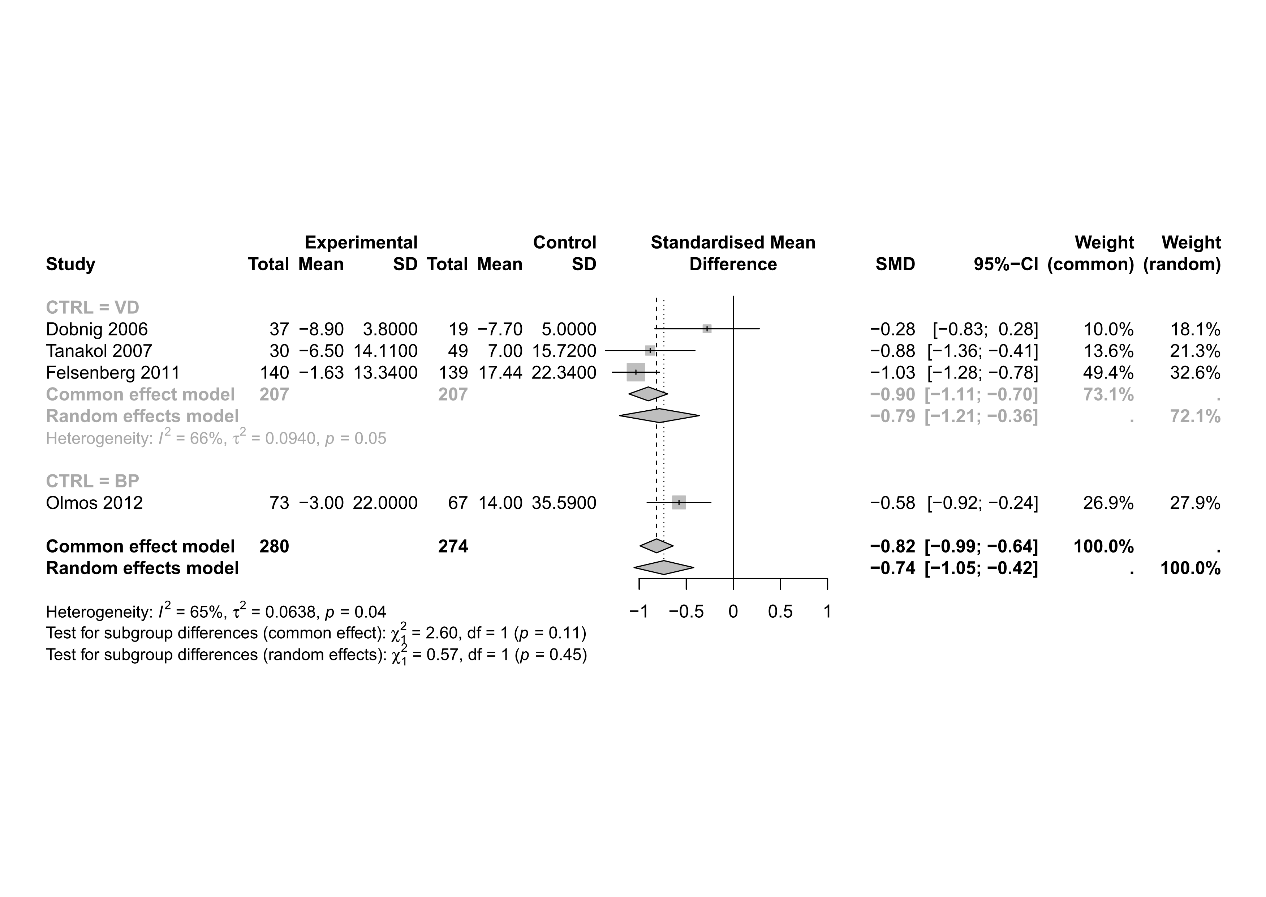


Figure S8 Subgroup analysis for PTH(per)


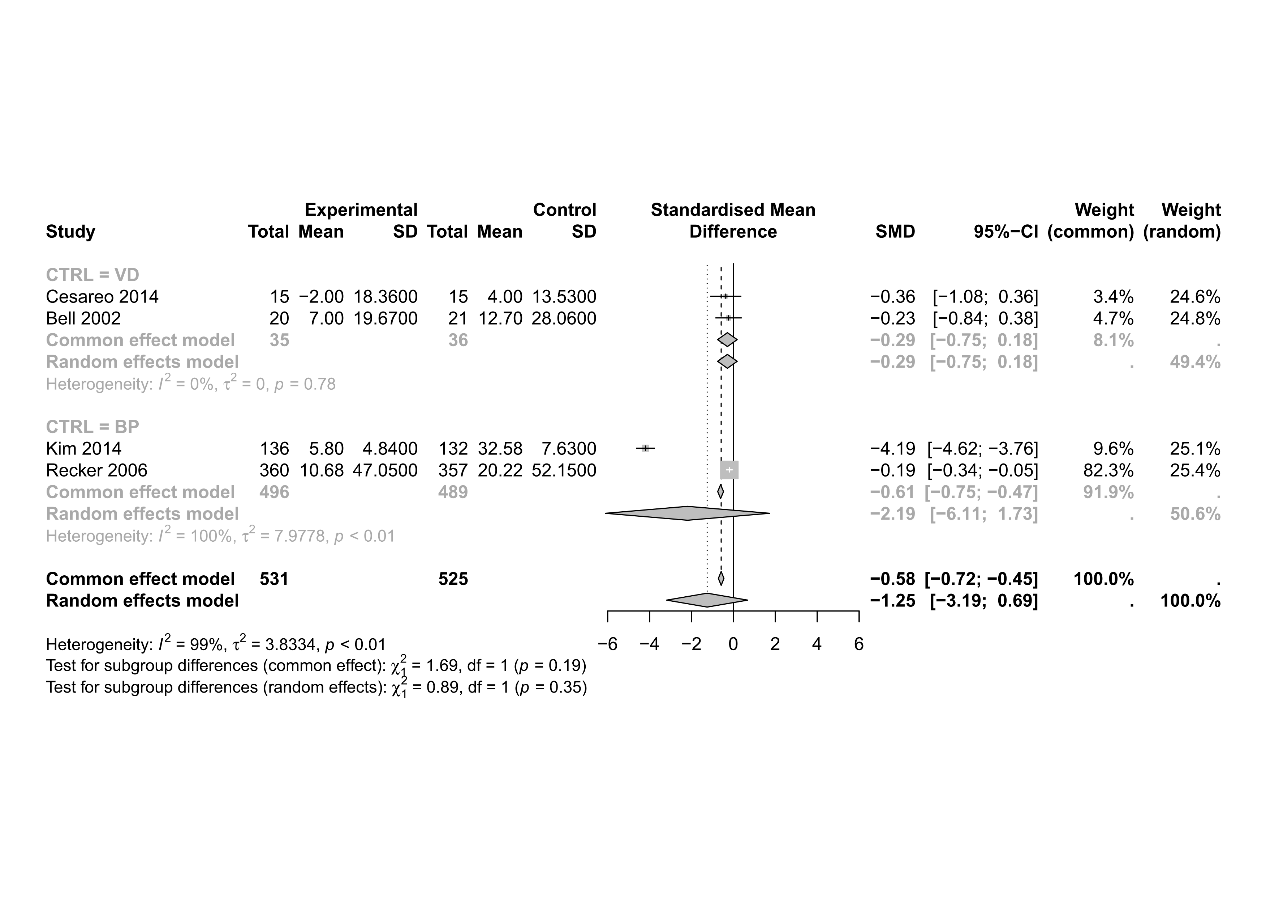


Figure S9 Subgroup analysis for sCa(abs)


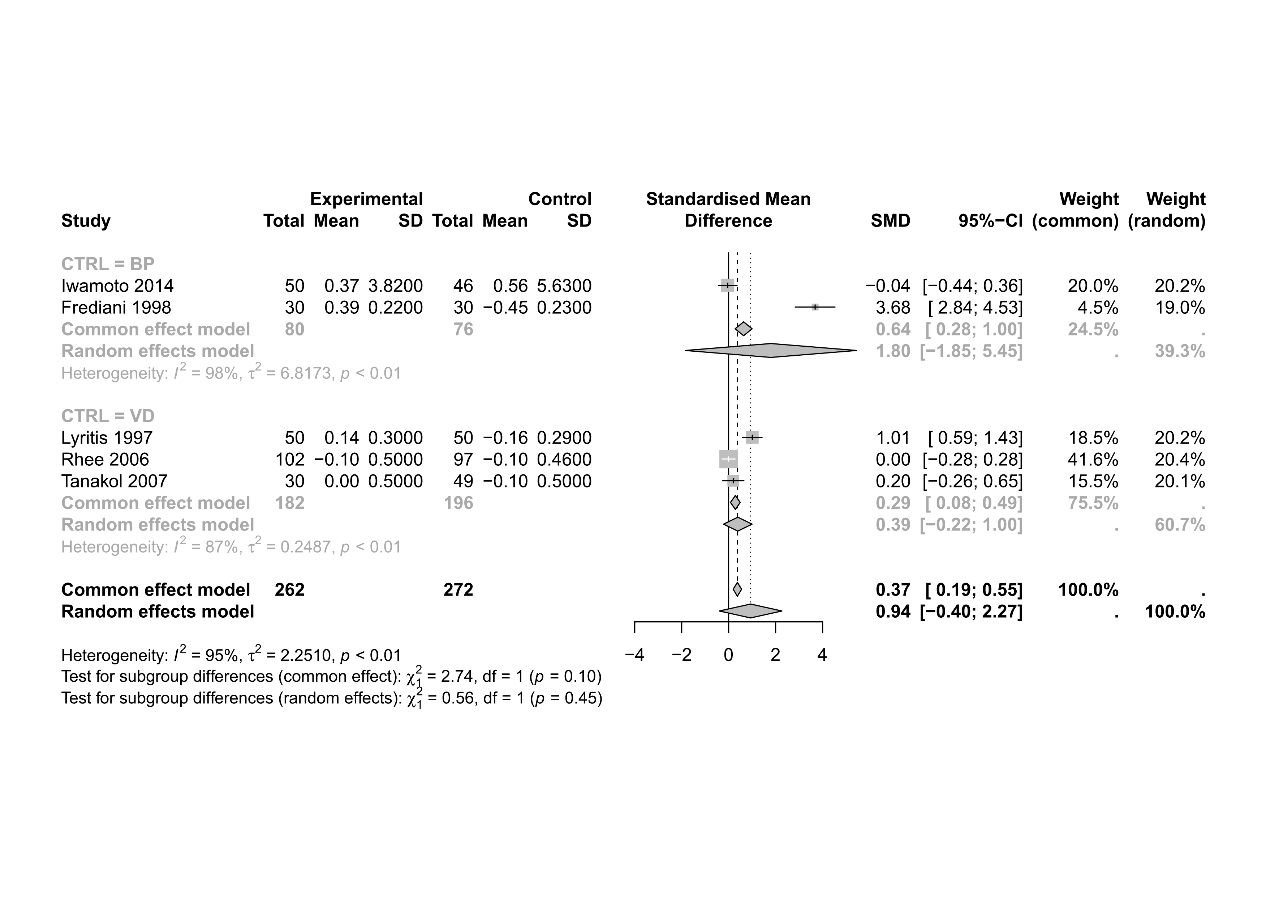


Figure S10 Subgroup analysis for AEs


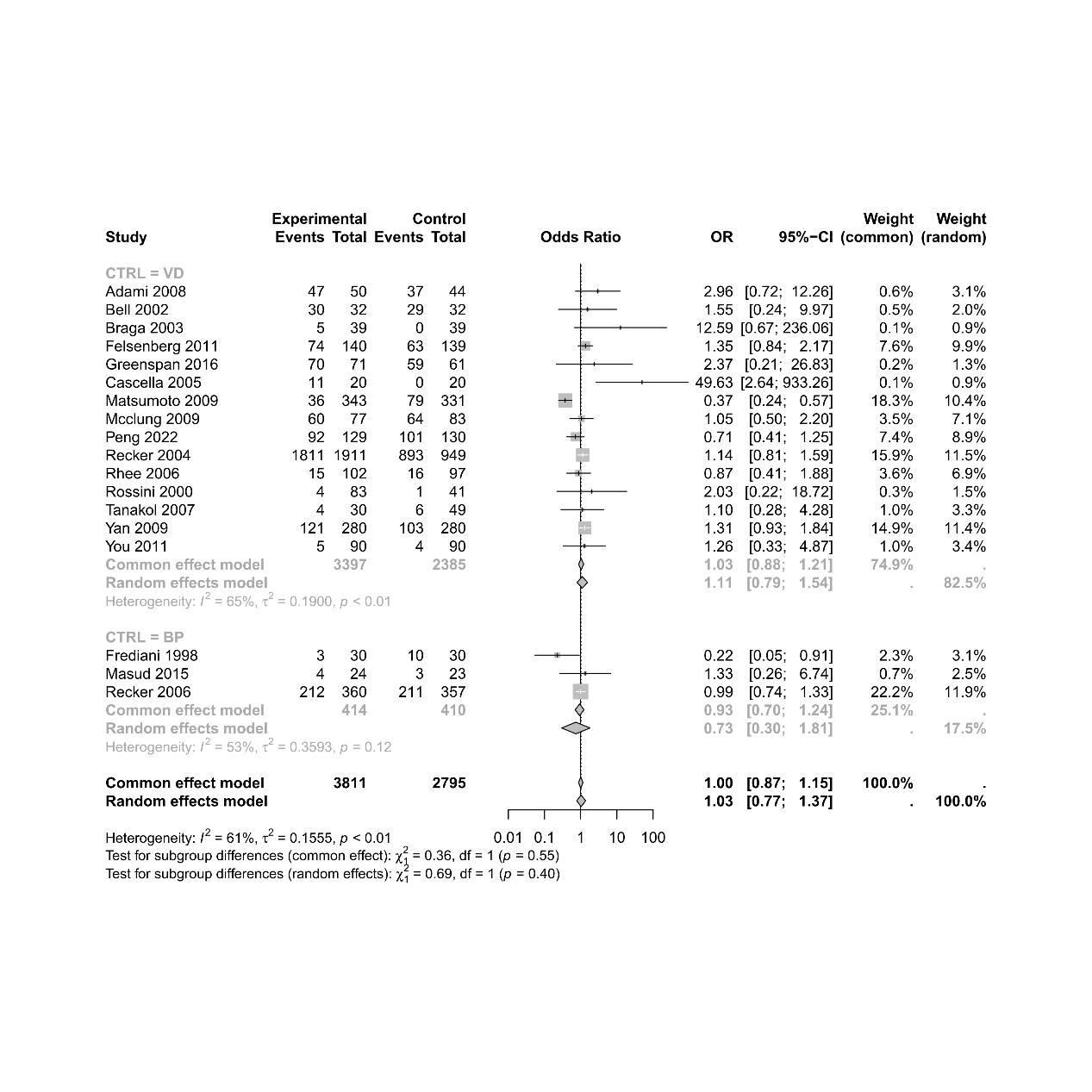


Figure S11 Subgroup analysis for SAEs


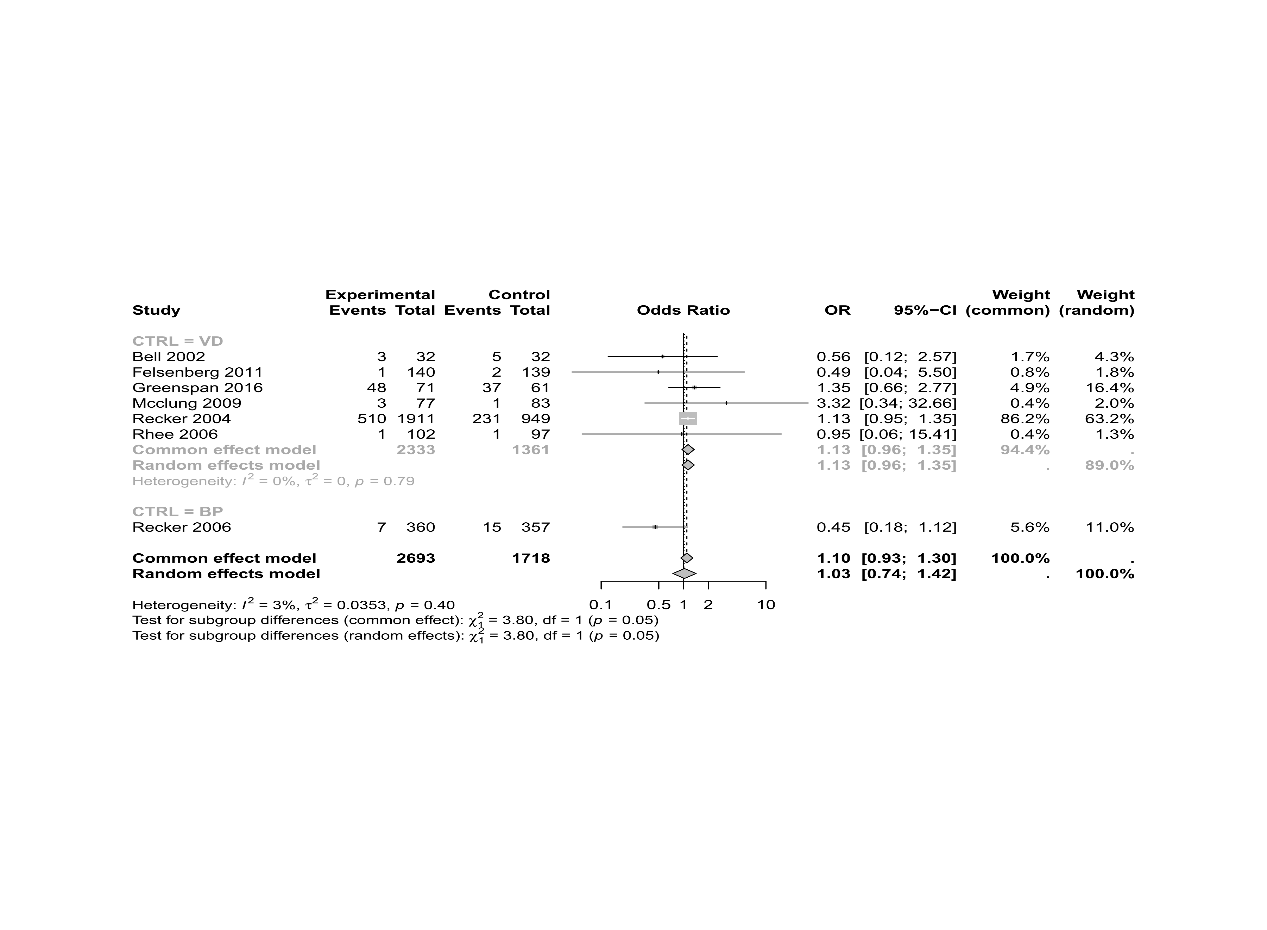


Figure S12 Subgroup analysis for sBALP(per)


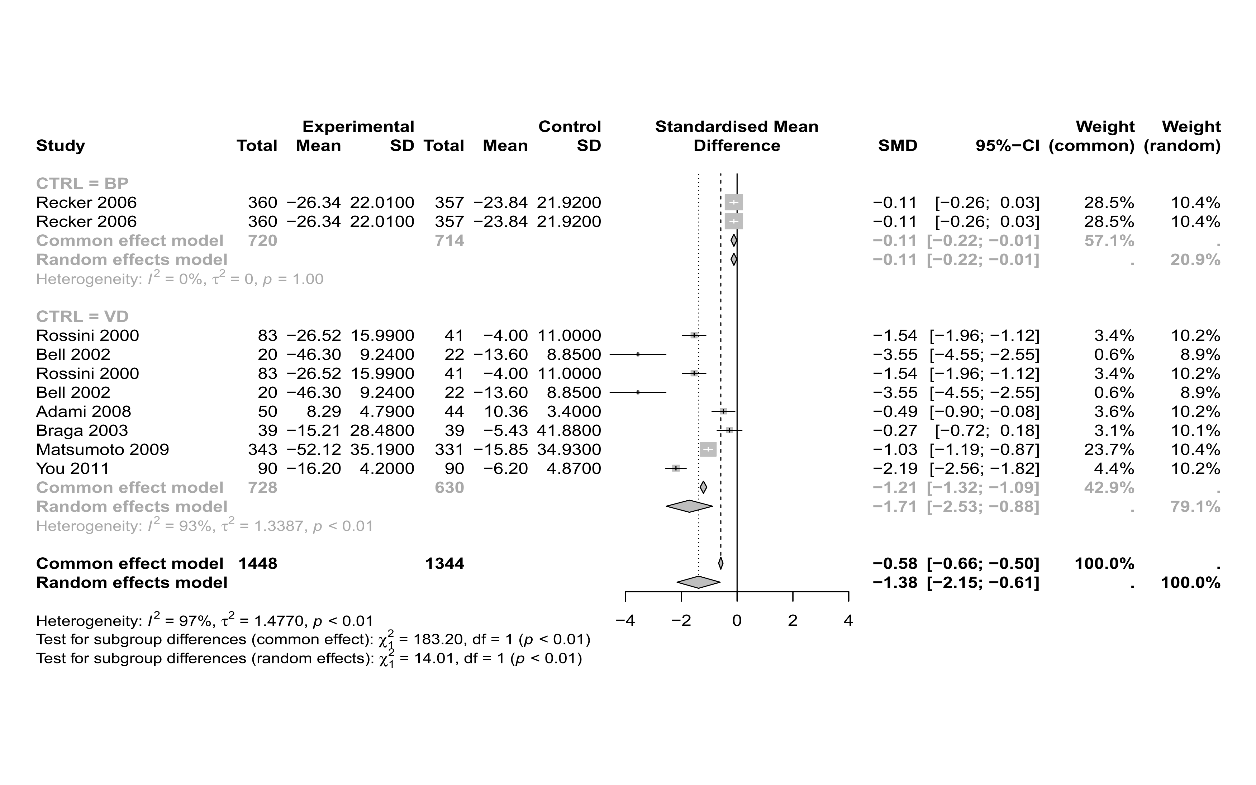


Figure S13 Subgroup analysis for sCTX(abs)


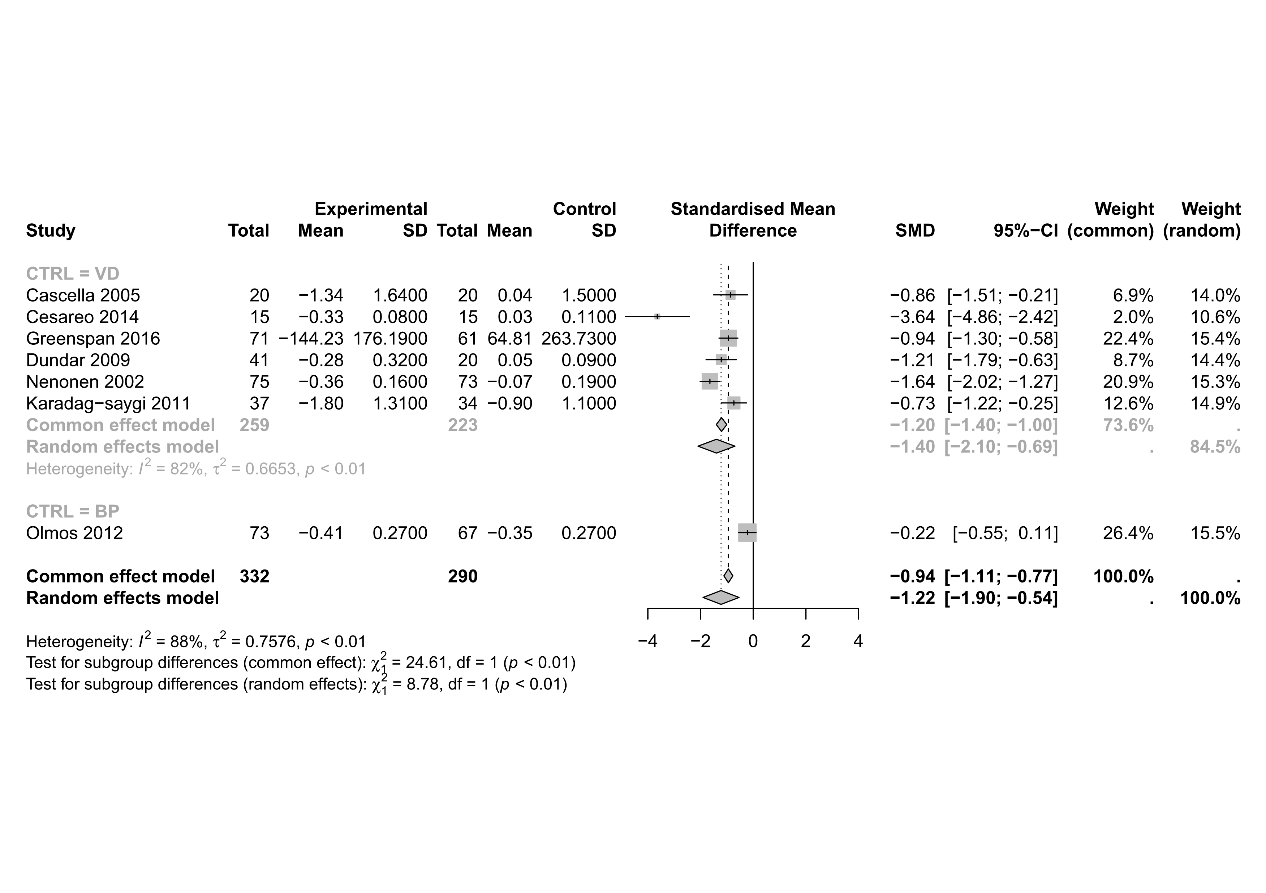


Figure S14 Subgroup analysis for Uri(per)


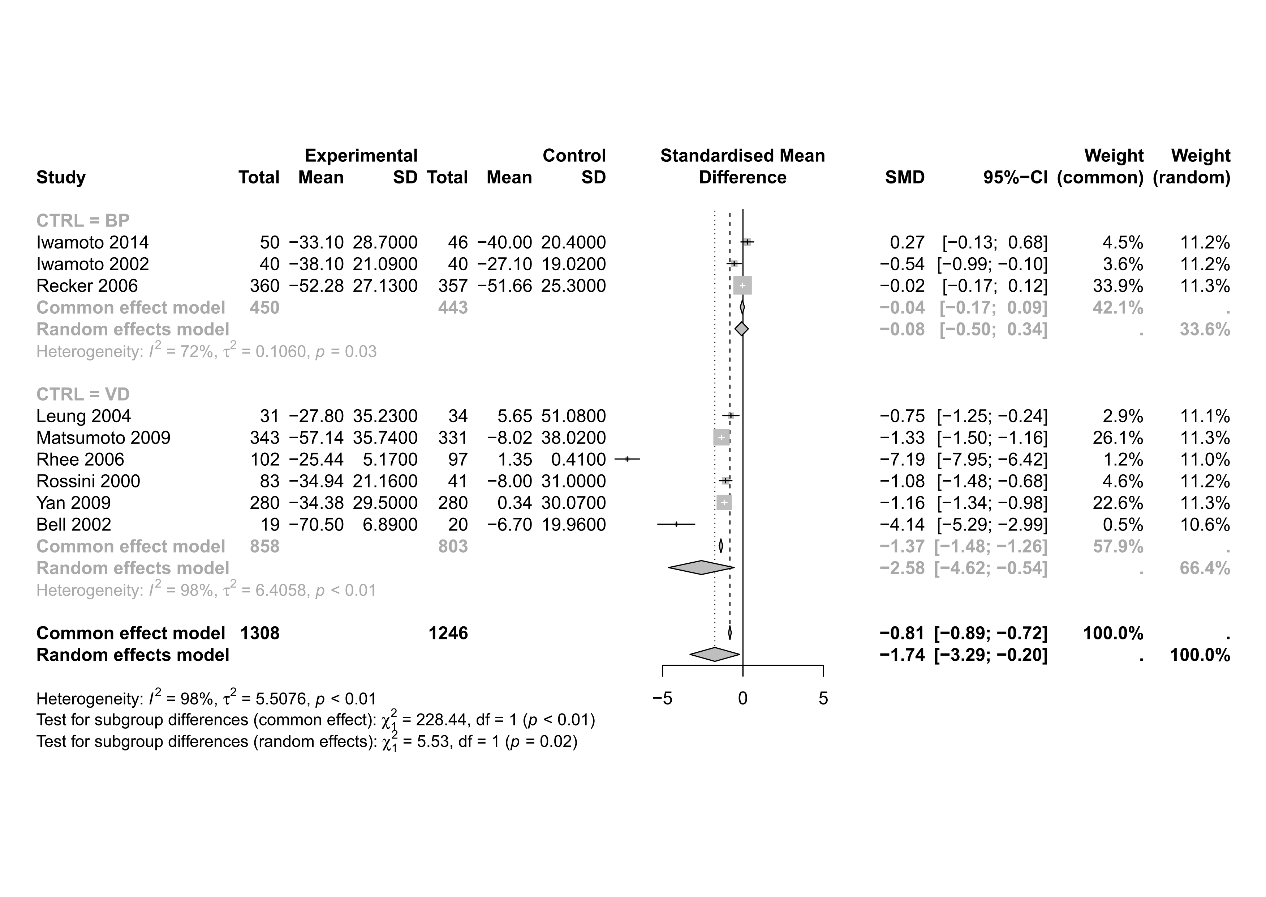


Figure S15 Sensitivity Analysis for 25-OH-VD(abs) changes.


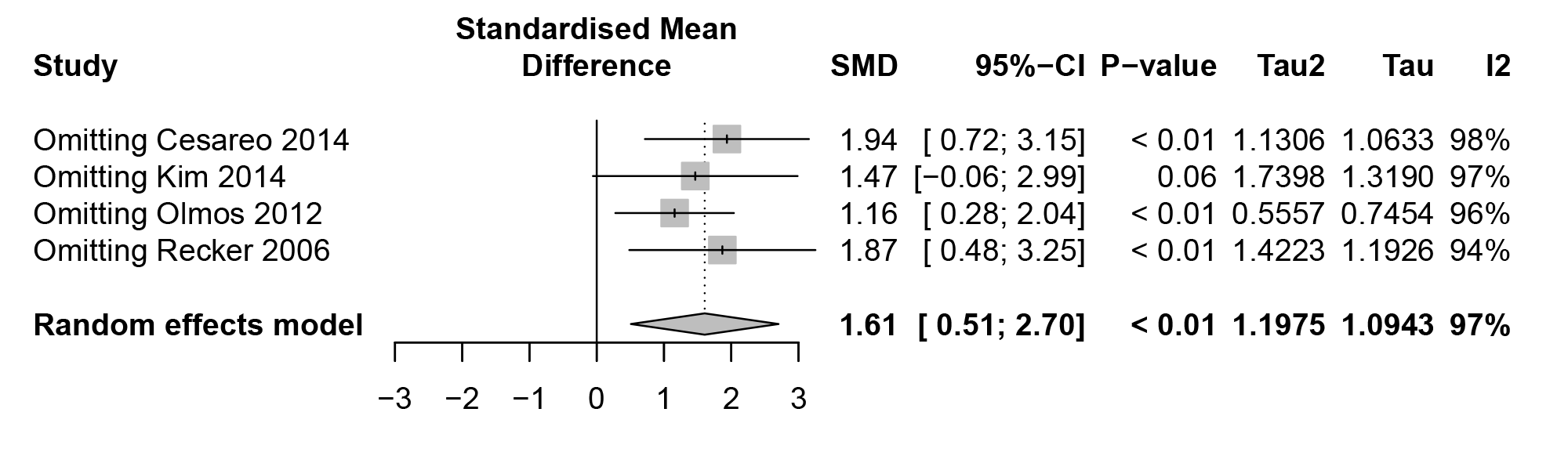


Figure S16 Sensitivity Analysis for Osteocalcin(abs) changes


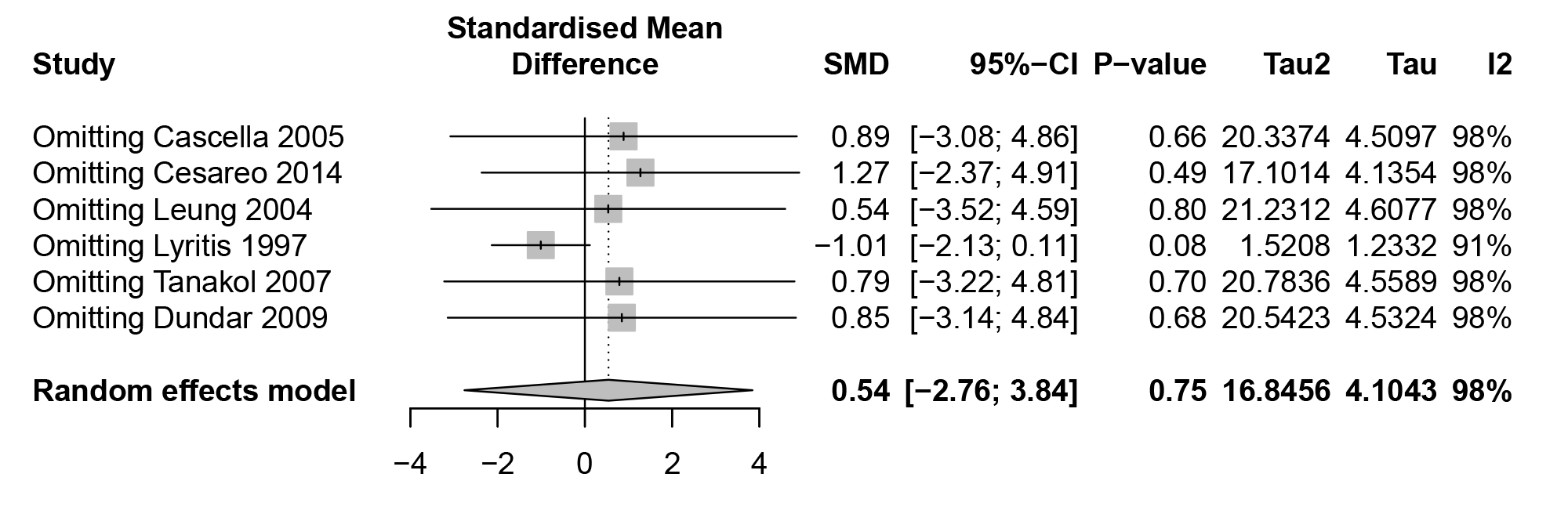


Figure S17 Sensitivity Analysis for PTH(abs) changes.


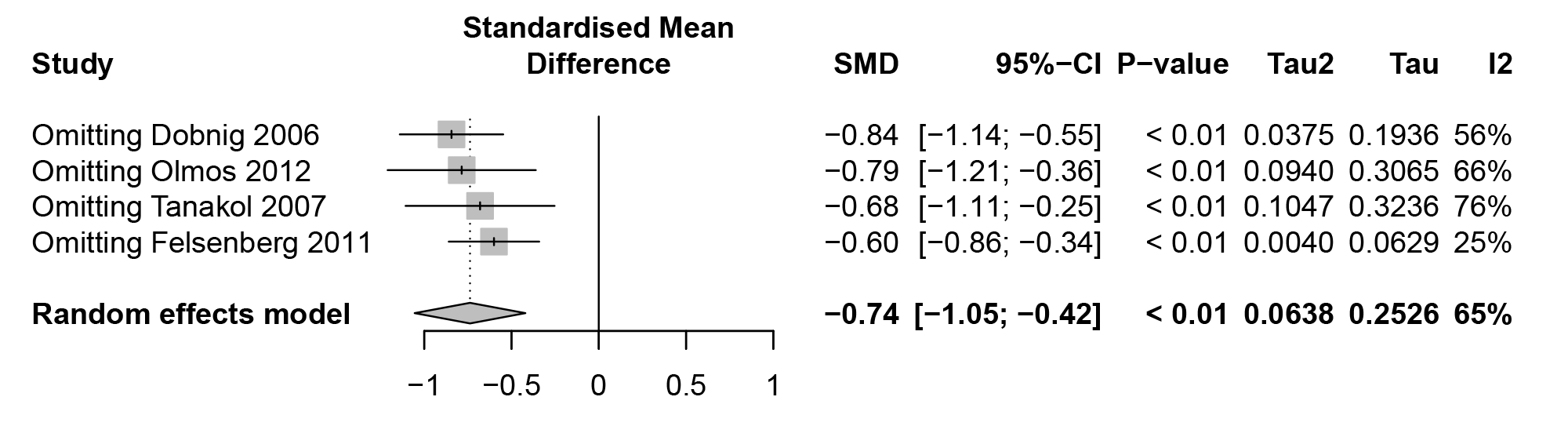


Figure S18 Sensitivity Analysis for sCa(abs) changes.


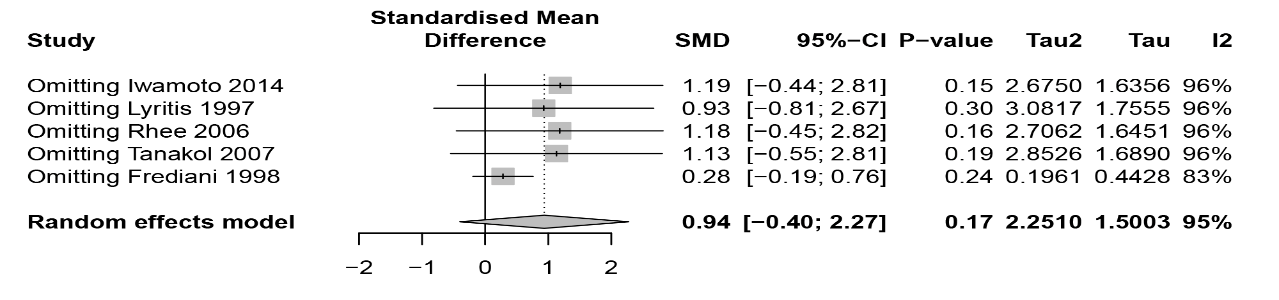


Figure S19 Sensitivity Analysis for sCTX(abs) changes.


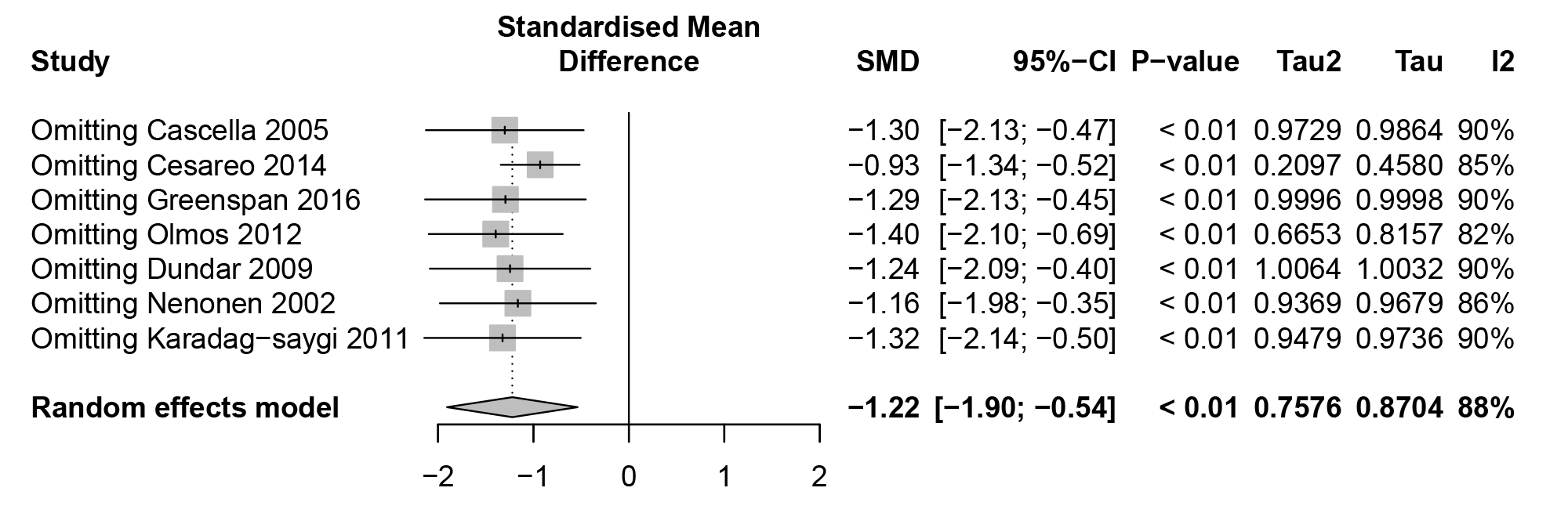


Figure S20 Sensitivity Analysis for sBALP(abs) changes.


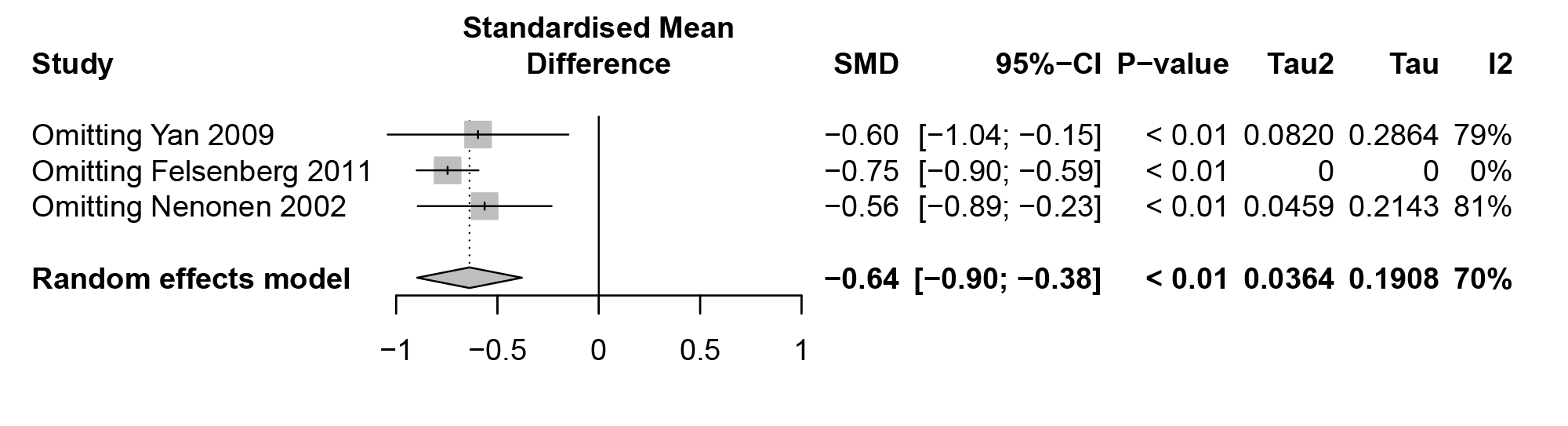


Figure S21 Sensitivity Analysis for fBMD changes.


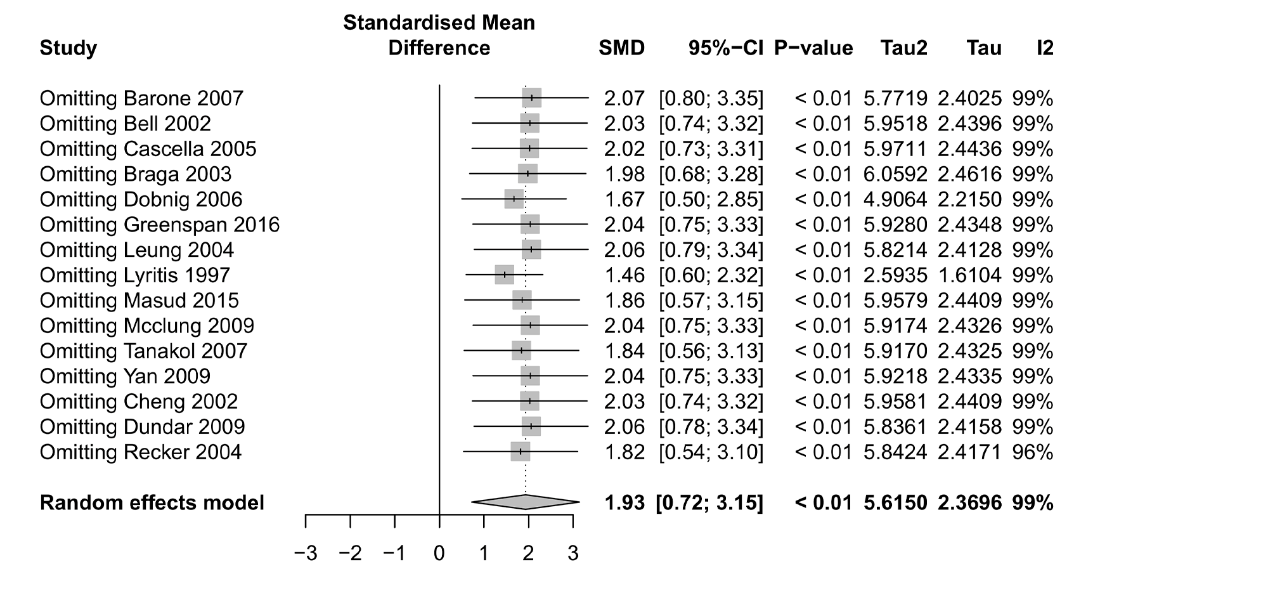


Figure S22 Sensitivity Analysis for ftroBMD changes.


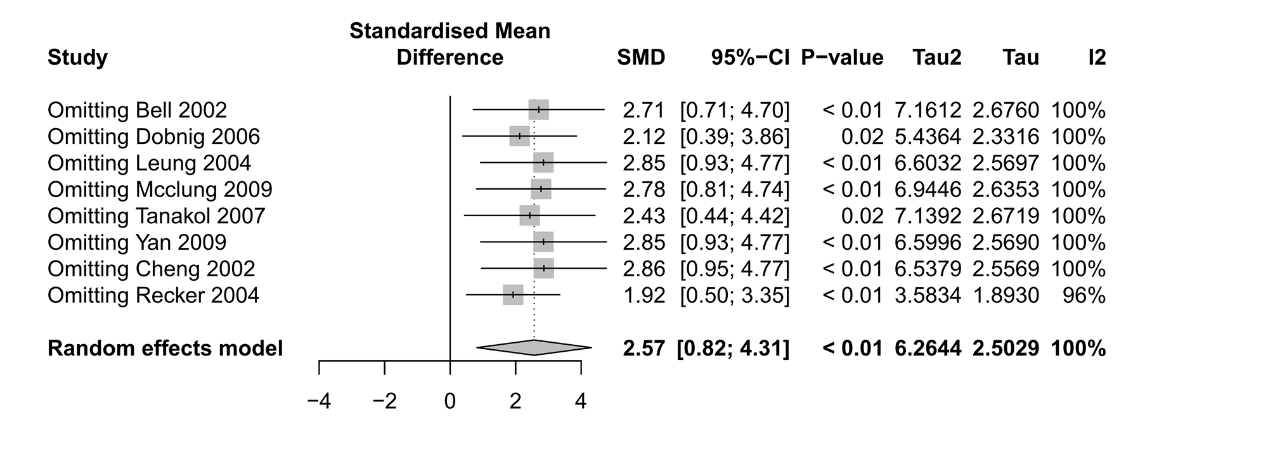


Figure S23 Sensitivity Analysis for LBMD changes.


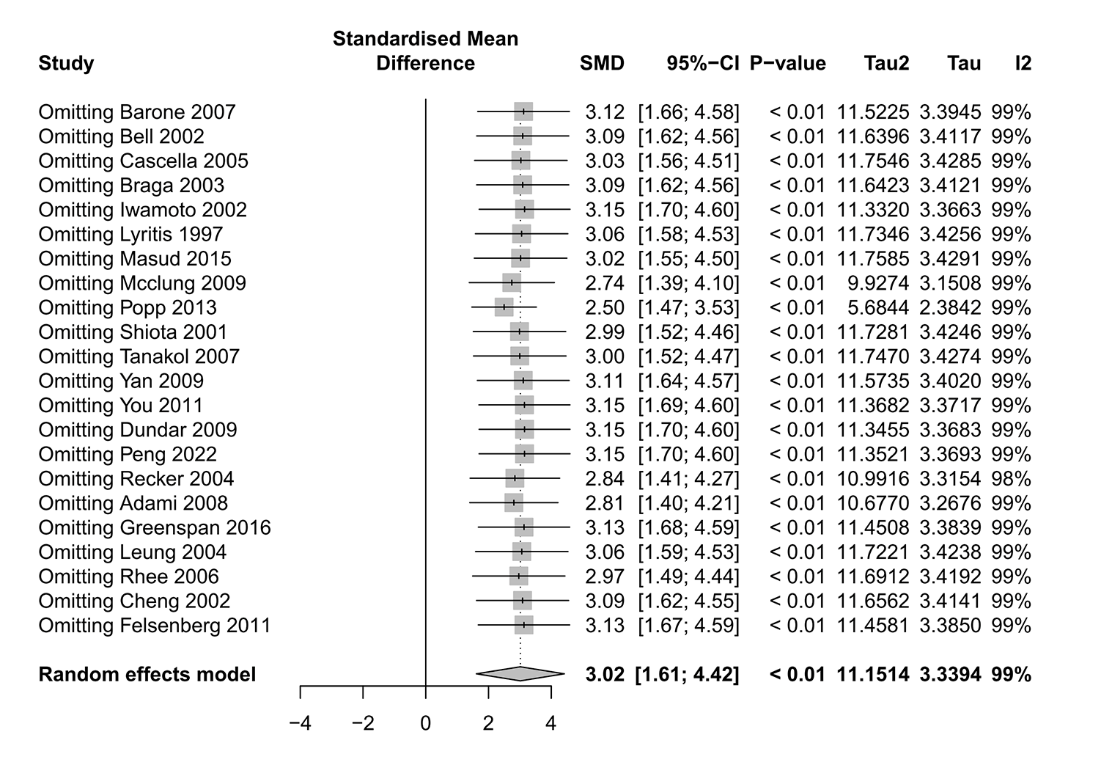


Figure S24 Sensitivity Analysis for 25-OH-VD(per) changes.


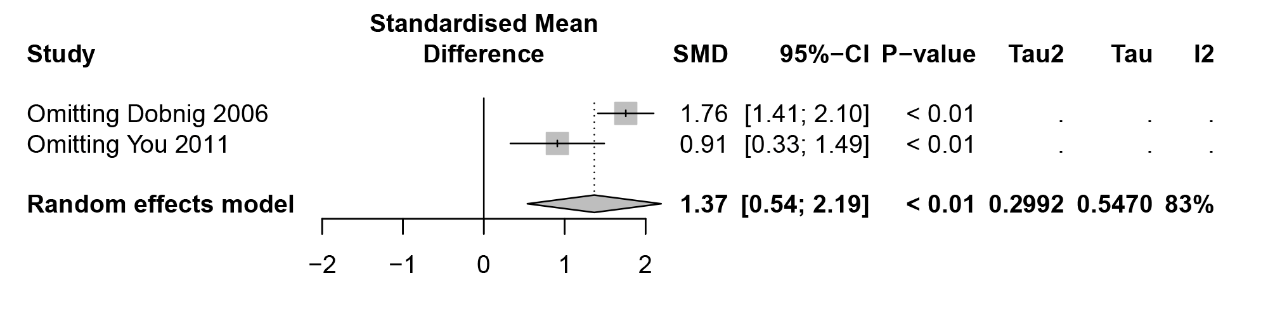


Figure S25 Sensitivity Analysis for Osteocalcin(per) changes.


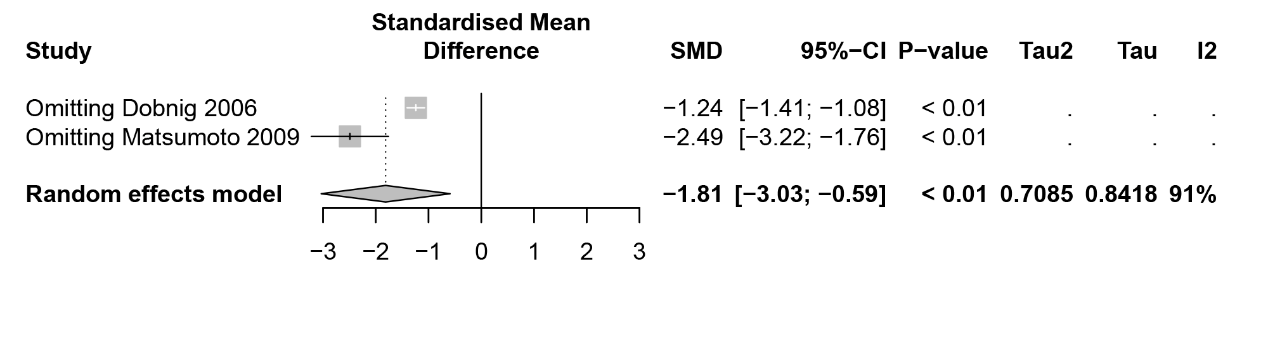


Figure S26 Sensitivity Analysis for PTH(per) changes.


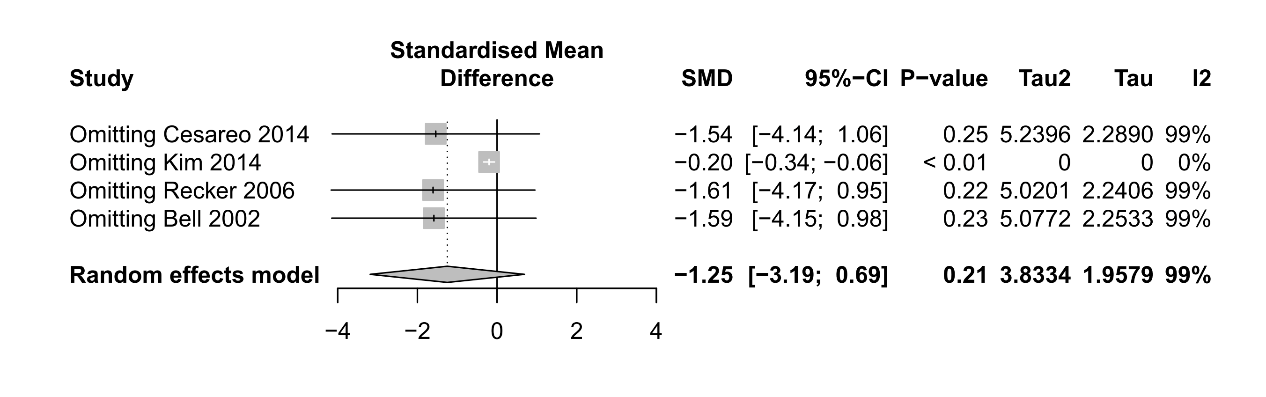


Figure S27 Sensitivity Analysis for sBALP(per) changes.


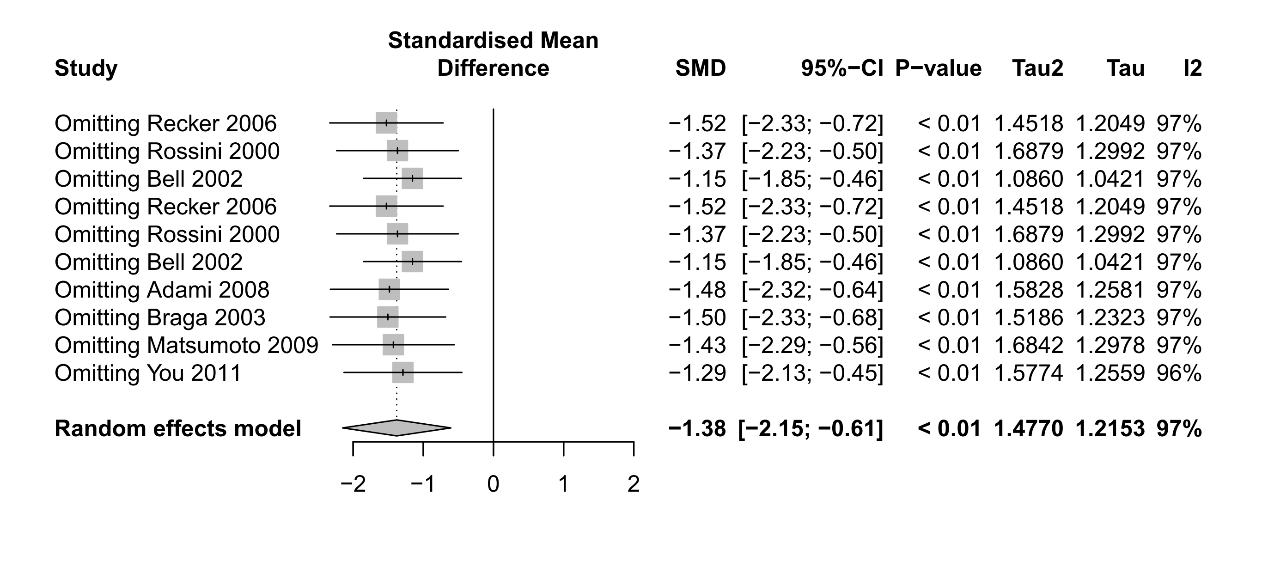


Figure S28 Sensitivity Analysis for sCTX(per) changes.


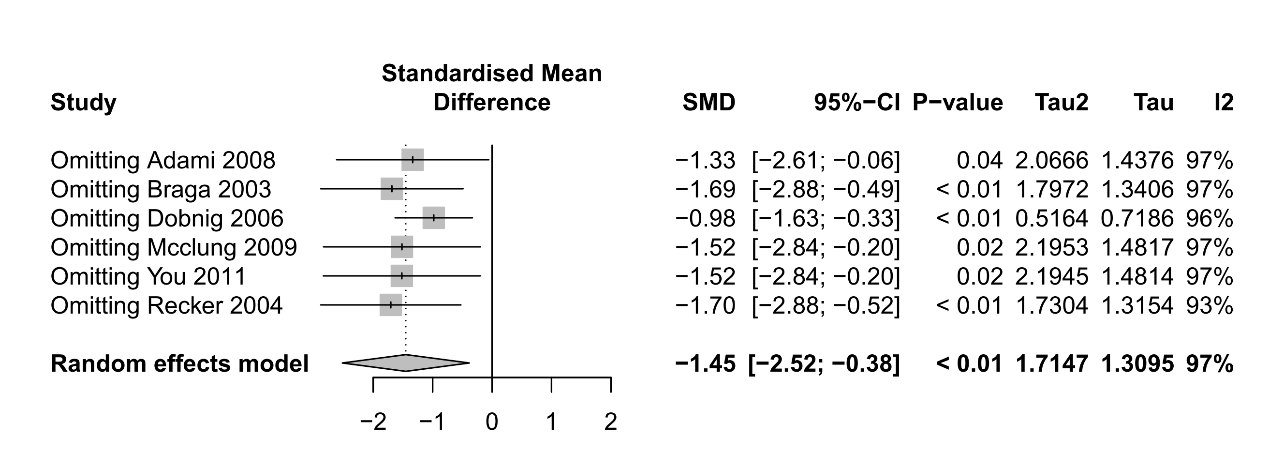


Figure S29 Sensitivity Analysis for uriNTX(per) changes.


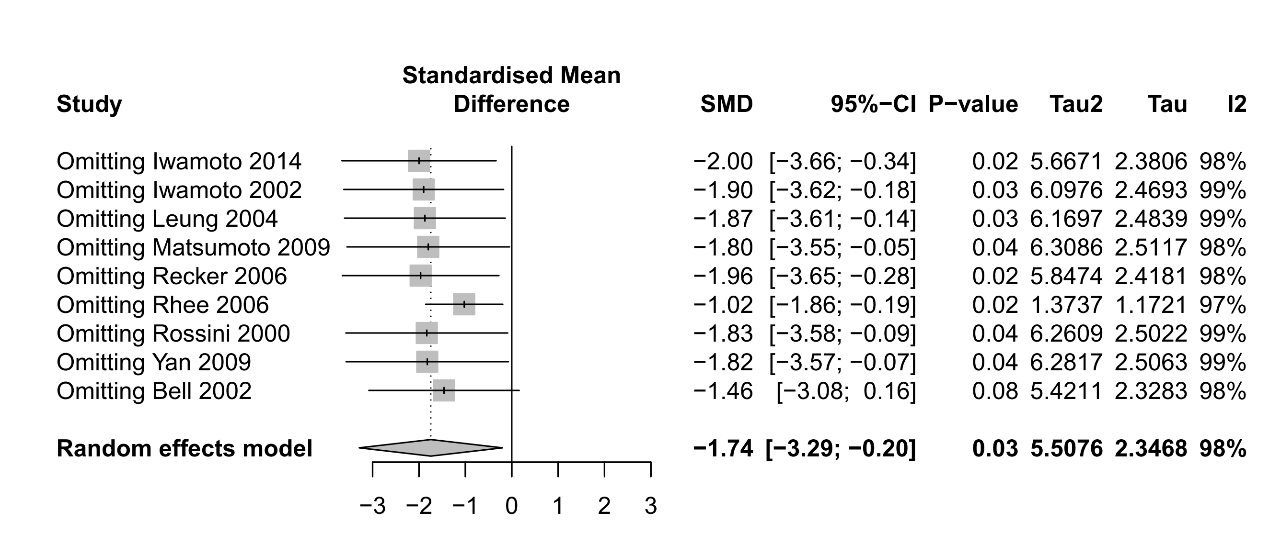


Figure S30 Sensitivity Analysis for ThipBMD changes.


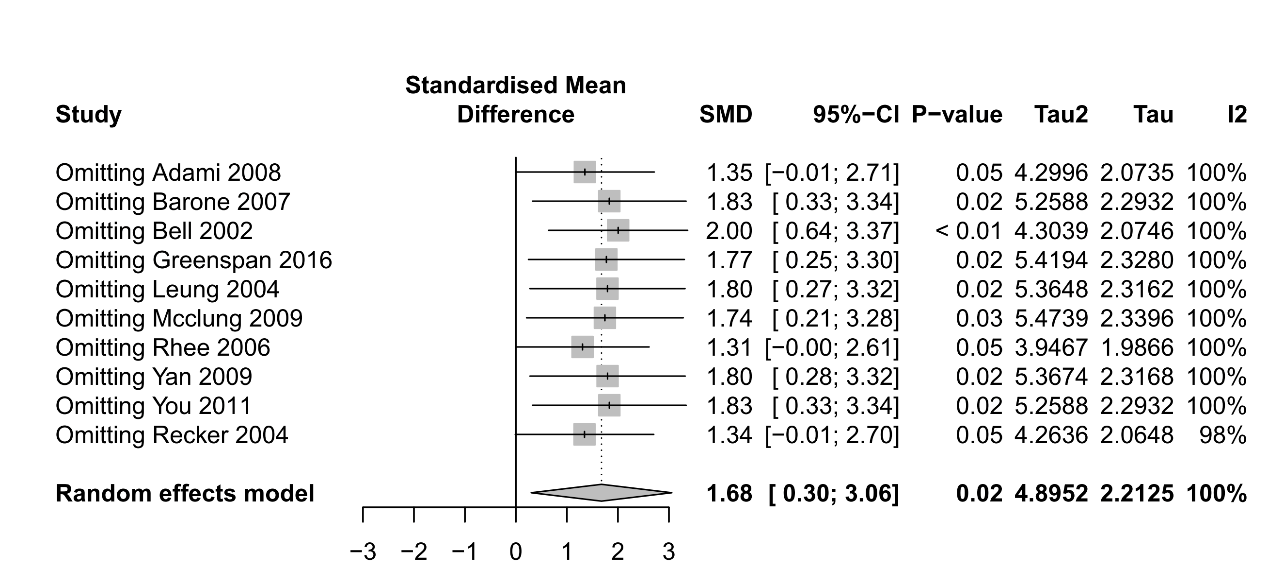


Figure S31 Sensitivity Analysis for ALP changes.


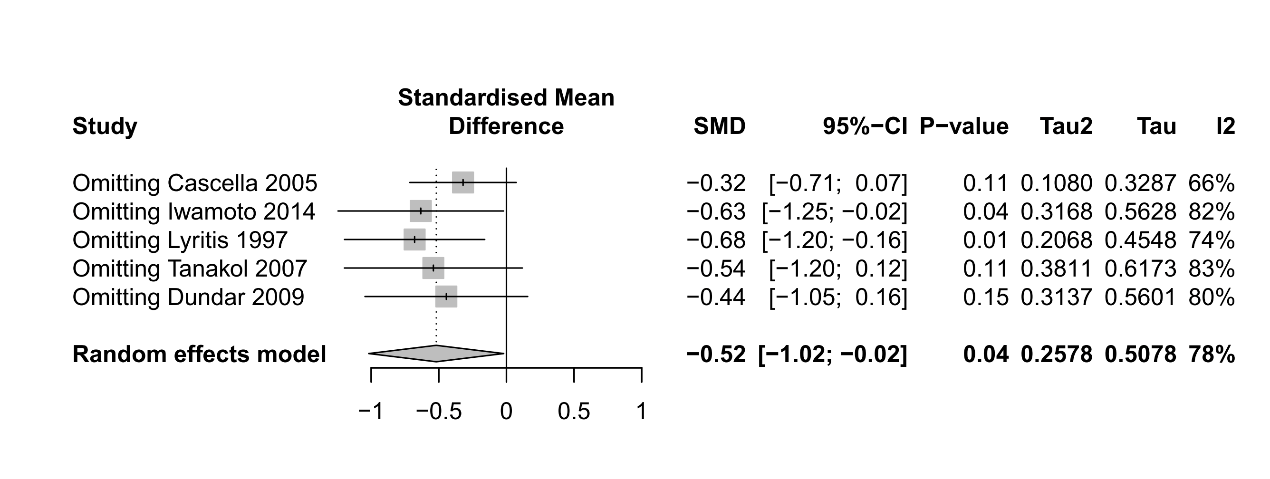


Figure S32 Sensitivity Analysis for AEs changes.


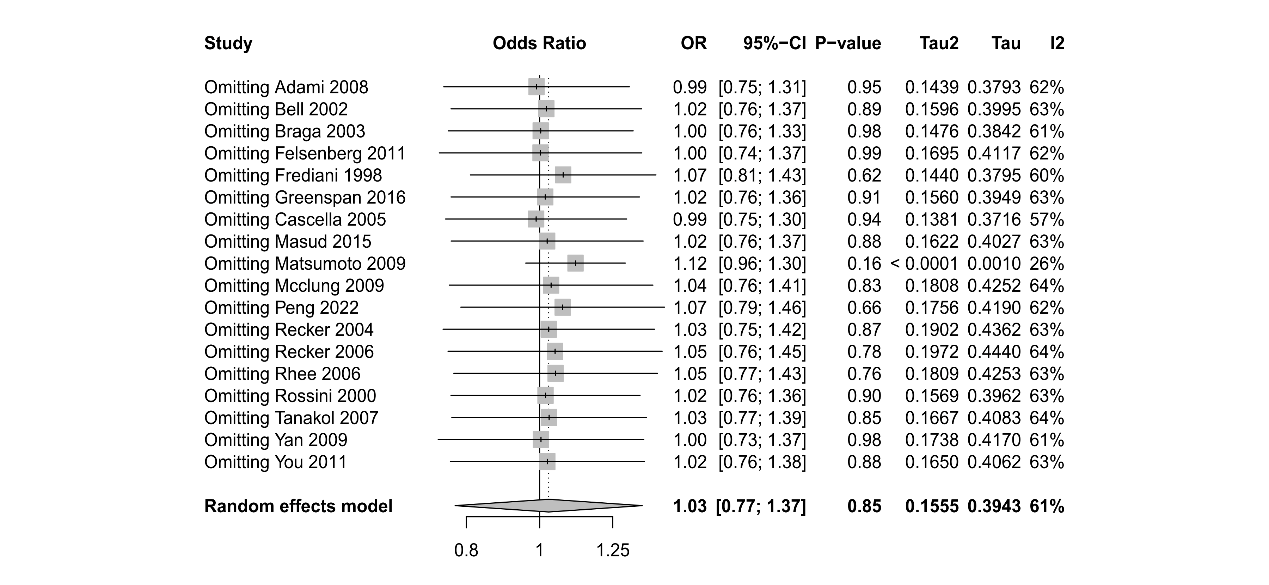


Figure S33 The publication bias for sBALP(per) changes.


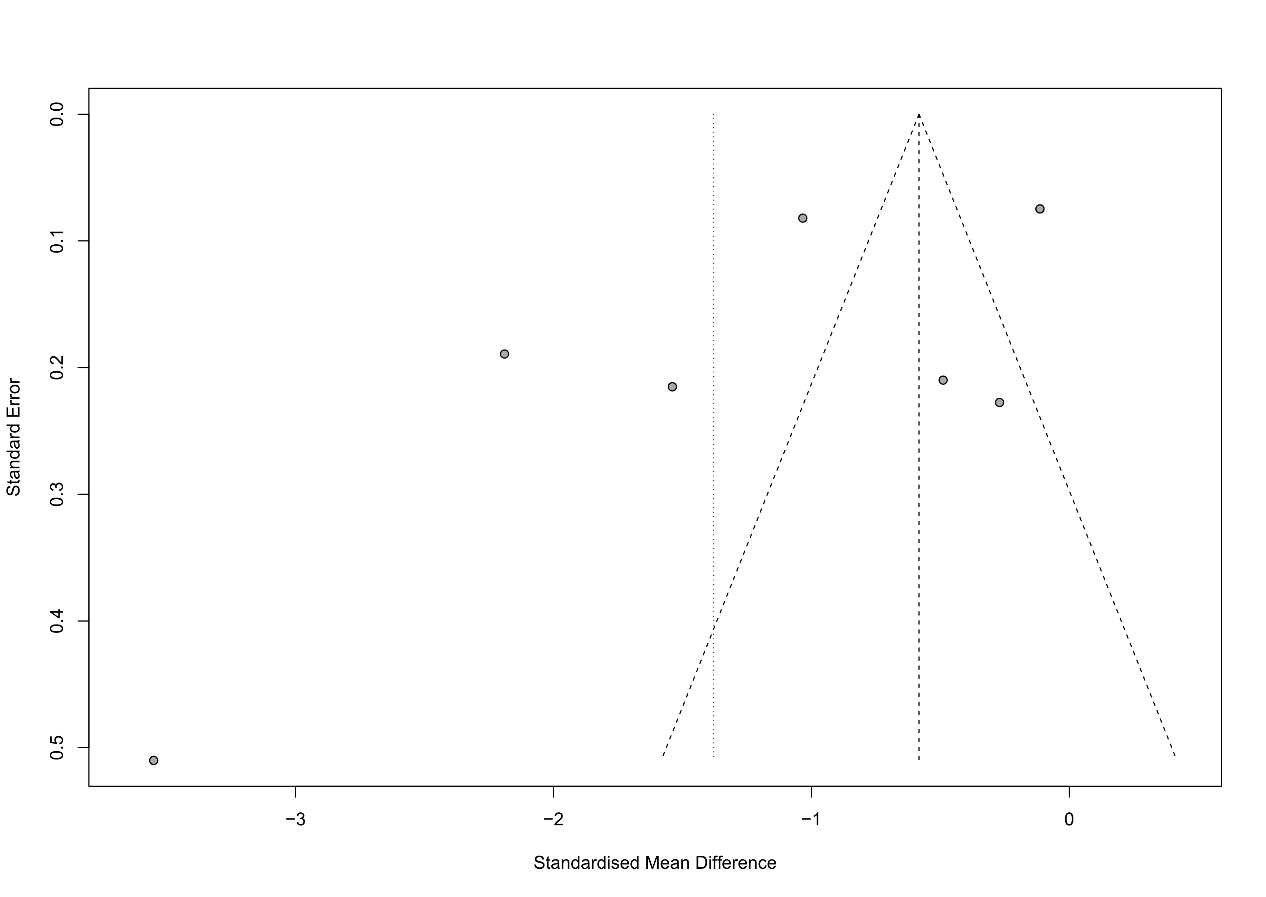


Figure S34 The publication bias for ThipBMD changes.


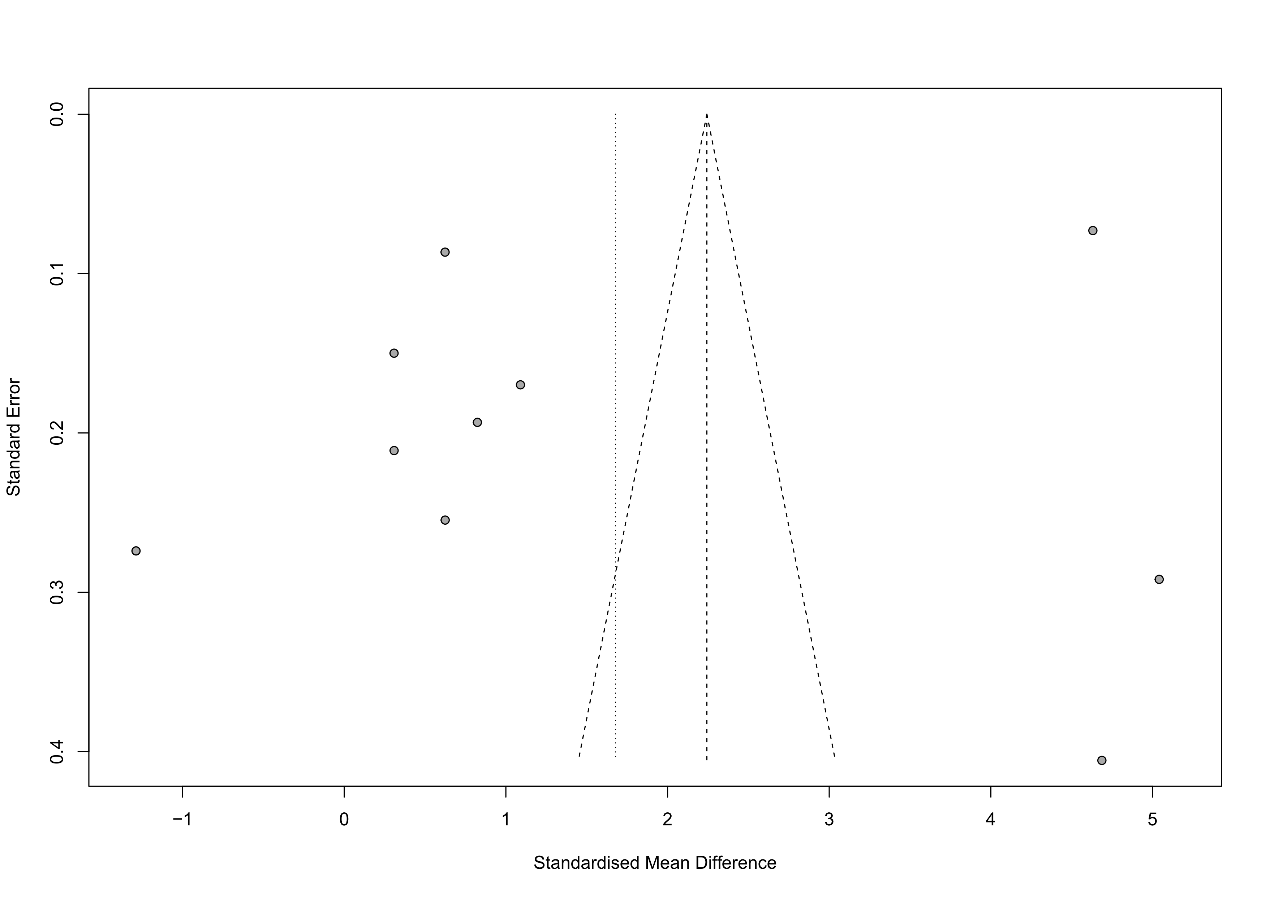


Figure S35 The publication bias for LBMD changes.


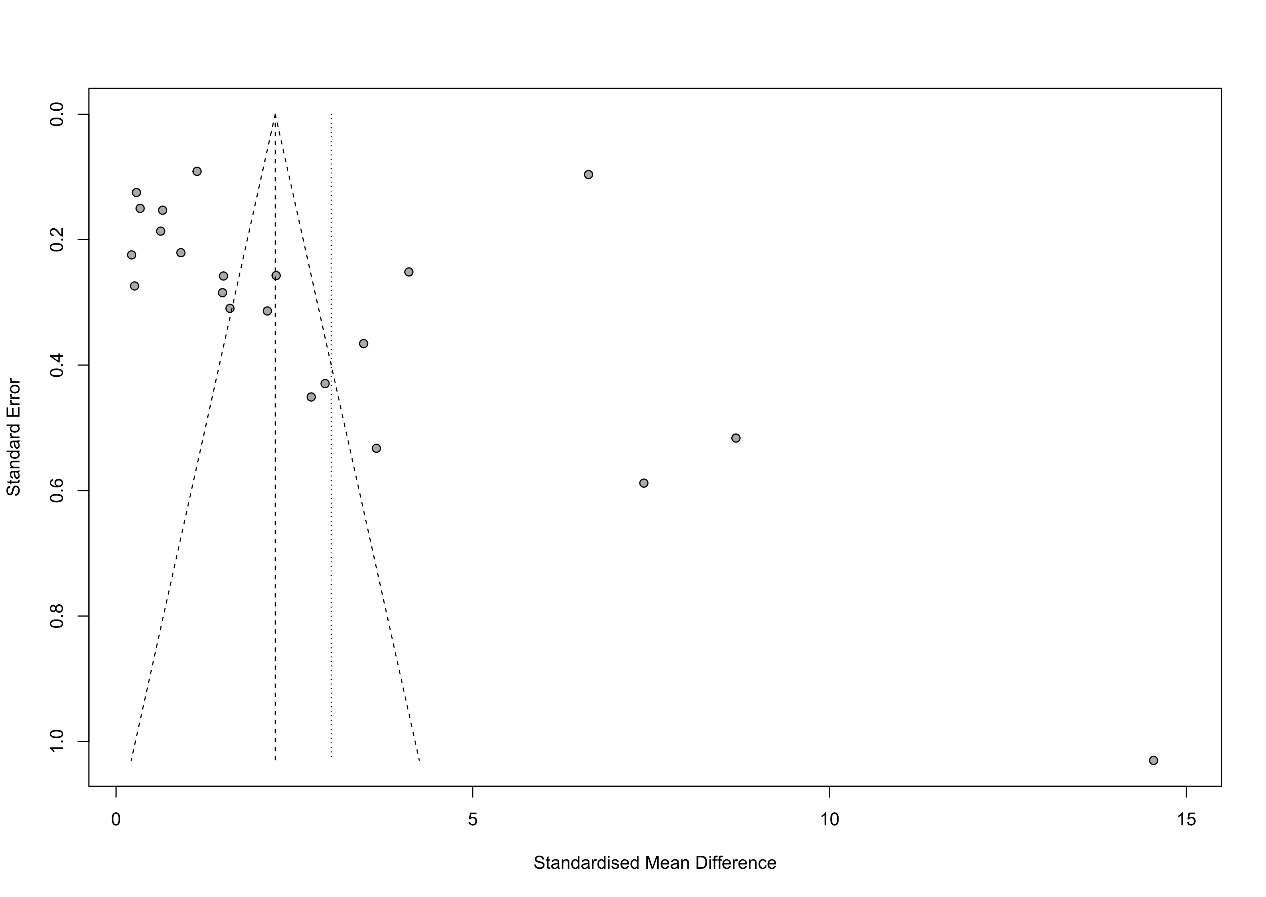


Figure S36 The publication bias for fBMD changes.


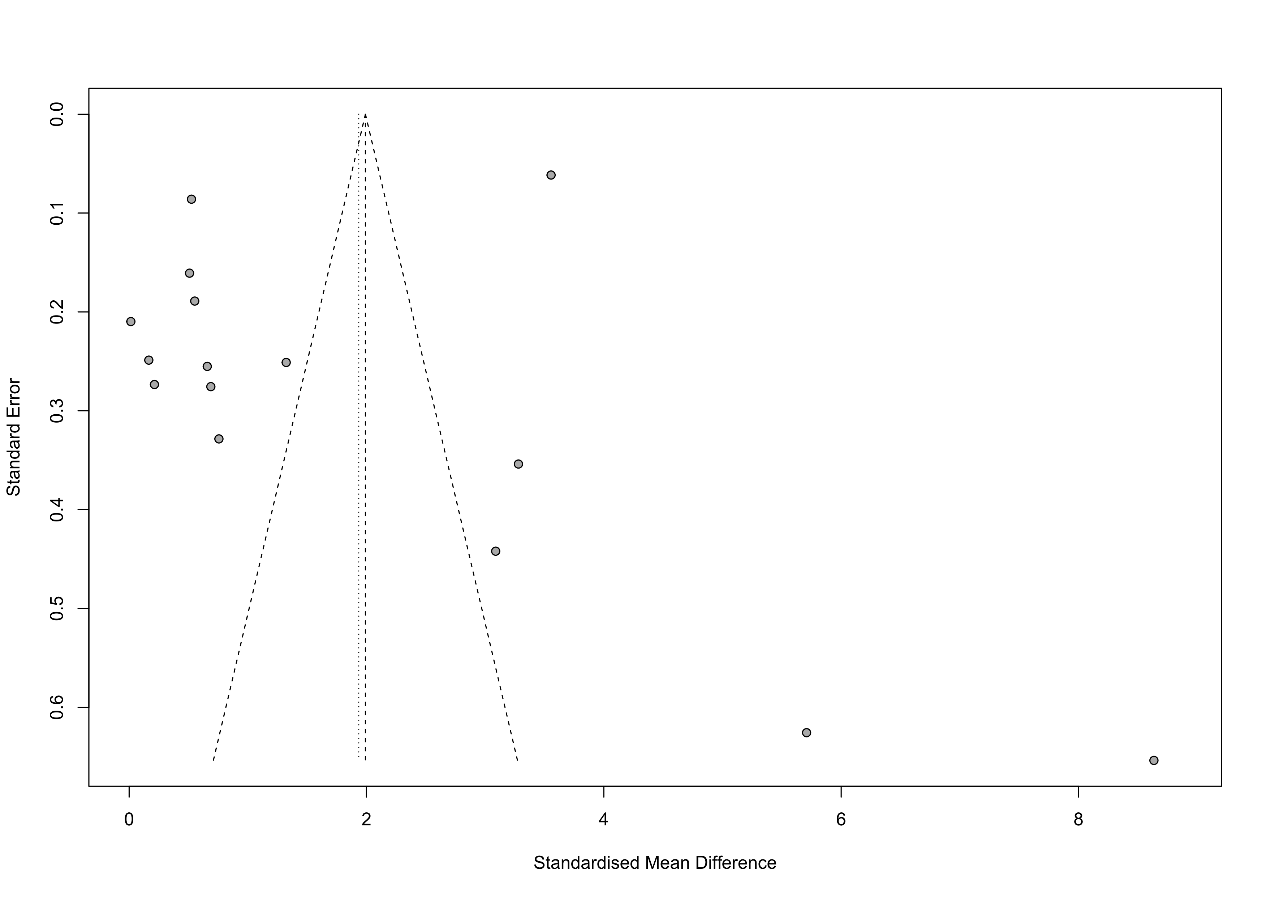


Figure S37 The publication bias for AEs changes.


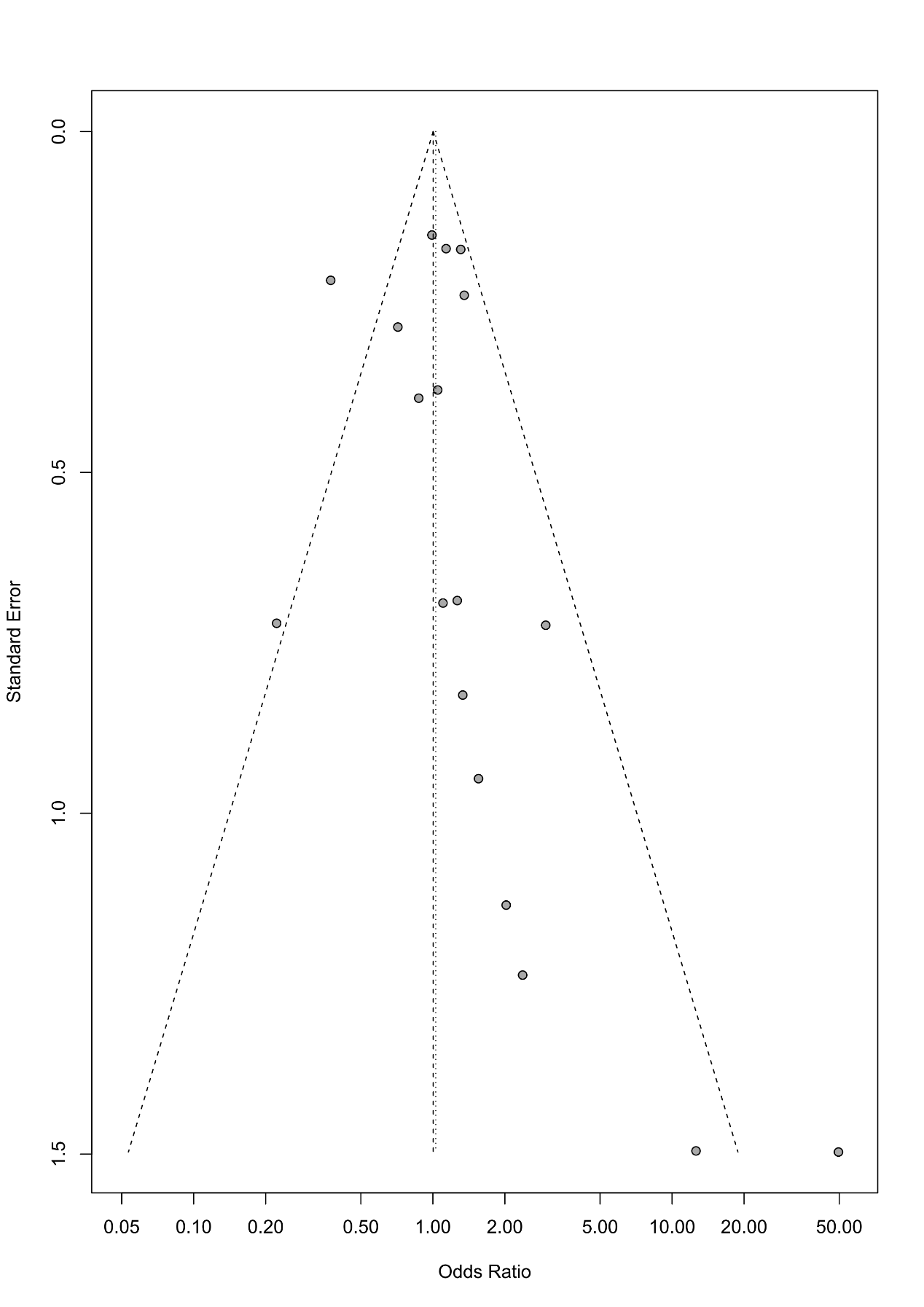


Figure S38 Results from the trim-and-fill method for publication bias in sBALP changes.


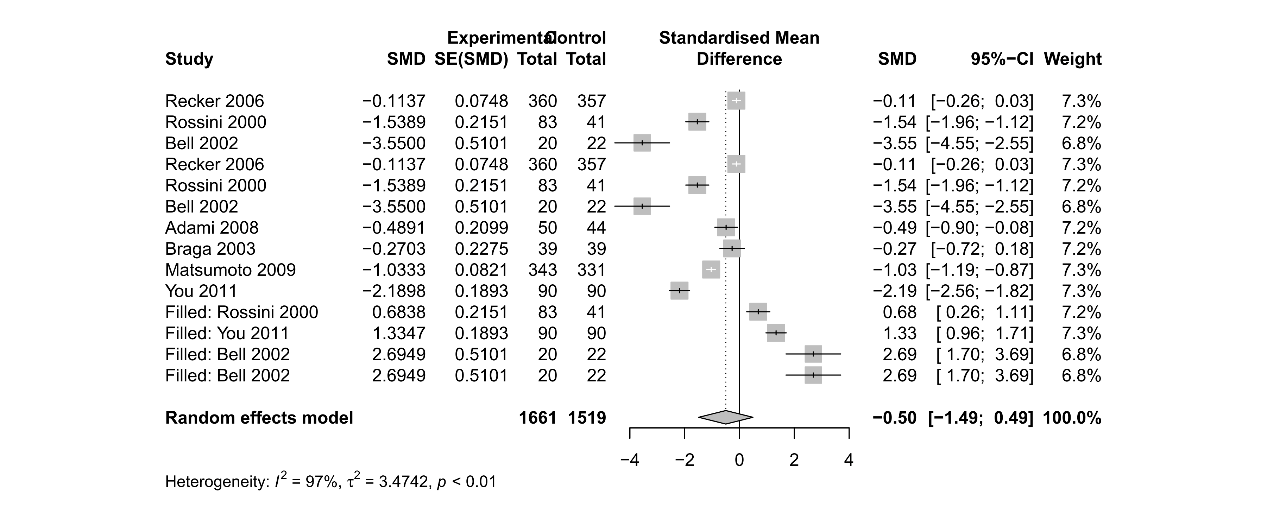

Supplement: Supplementary file 1 [file DataSheet1.zip › Supplementary material 1.DOCX]
