# Supplementary material for: Efficacy of combination therapy of vitamin D and bisphosphonates in the treatment of postmenopausal osteoporosis: a systematic review and meta-analysis
Source: Front Pharmacol. 2024 Nov 21;15:1422062. doi: 10.3389/fphar.2024.1422062 (PMC11617160; doi:10.3389/fphar.2024.1422062)
Supplement: Supplementary file 1 [file DataSheet1.zip › Supplementary material 3.DOCX]

**Supplementary material 3** Meta-analysis

| Outcome | Trials | Sample size | Heterogeneity | Pooled estimation | Sensitivity analysis | Subgroup analysis | Publication bias test  Egger- P |
| --- | --- | --- | --- | --- | --- | --- | --- |
| LBMD | 22 | 5522 | I^2^=99.40%  p=0 | 3.02  [1.61, 4.42]  p< 0.01 | stable | P=0.08  No significant difference | 0.85 |
| ftroBMD | 8 | 3901 | I^2^=99.70%  p=0 | 2.57  [0.82,4.31] p=0.04 | stable | - | - |
| fBMD | 15 | 4435 | I^2^=99.10%  p<0.01 | 1.93  [0.72, 3.15] p=0.01 | stable | p=0.78  No significant difference | 0.31 |
| ThipBMD | 10 | 4391 | I^2^=99.60%  p=0 | 1.68  [0.30,3.06] p=0.02 | stable | p=0.06  No significant difference | 0.31 |
| ALP | 5 | 376 | I^2^=78.40%  p=0.01 | -0.52  [-1.02, -0.02] p=0.04 | stable | P=0.18  No significant difference | - |
| sBALP abs | 3 | 987 | I^2^=70.30%  p=0.03 | -0.64  [-0.8962, -0.3793] p< 0.01 | stable | - | - |
| sBALP per | 10 | 2792 | I^2^=96.90%  p<0.01 | -1.3782  [-2.1494, -0.6071]  p=0.01 | stable | P<0.01  significant difference | 0.02 |
| Osteocalcin abs | 6 | 375 | I^2^=97.90%  p<0.01 | 0.54  [-2.76,3.84] p=0.75 | stable | - | - |
| Osteocalcin per | 2 | 730 | I^2^=90.70%  p=0.01 | -1.81  [-3.03, -0.59] p=0.01 | stable | - | - |
| PTH abs | 4 | 554 | I^2^=64.70%  p=0.04 | -0.74  [-1.05,-0.42]  p< 0.01 | stable | P=0.45  No significant difference | - |
| PTH per | 4 | 1056 | I^2^=99.00%  p<0.01 | -1.25  [-3.19, 0.69] p=0.21 | stable | P=0.35  No significant difference | - |
| 25-OH-VD abs | 4 | 1155 | I^2^=97.10%  p<0.01 | 1.61  [0.51,2.70] p=0.01 | stable | P=0.05  No significant difference | - |
| 25-OH-VD per | 2 | 236 | I^2^=83.50%  p=0.01 | 1.37  [0.54,2.19] p=0.01 | stable | - | - |
| sCa abs | 5 | 534 | I^2^=94.90%  p<0.01 | 0.94  [-0.40,2.27] p=0.17 | stable | P=0.45  No significant difference | - |
| sCa per | 1 | 180 | - | 2.99  [2.56,3.41]  p< 0.01 | - | - | - |
| sCTX abs | 7 | 622 | I^2^=88.50%  p<0.01 | -1.22  [-1.90,-0.54] p=0.01 | stable | P<0.01  significant difference | - |
| sCTX per | 6 | 3428 | I^2^=96.80%  p<0.01 | -1.45  [-2.52,-0.38] p=0.01 | stable | - | - |
| UriNTX abs | 1 | 279 | - | -0.23  [-0.47,0.01] p=0.06 | - | - | - |
| UriNTX per | 9 | 2554 | I^2^=98.40%  p<0.01 | -1.74  [-3.29,0.20] p=0.03 | stable | P=0.02  significant difference | - |
| AE | 18 | 6606 | I^2^=61.00%  p<0.01 | 1.03  [0.77,1.37] p=0.85 | stable | P=0.40  No significant difference | 0.28 |
| SAE | 7 | 4411 | I^2^=3.00%  P=0.40 | 1.10  [0.93,1.30] p=0.28 |  | P=0.05  No significant difference | - |
