# Supplementary material for: Efficacy of combination therapy of vitamin D and bisphosphonates in the treatment of postmenopausal osteoporosis: a systematic review and meta-analysis
Source: Front Pharmacol. 2024 Nov 21;15:1422062. doi: 10.3389/fphar.2024.1422062 (PMC11617160; doi:10.3389/fphar.2024.1422062)
Supplement: Supplementary file 1 [file DataSheet1.zip › Supplementary material 2.DOCX]

**Supplementary material 2 Search Strategy**

**1.cochrane**

Search Name:

Date Run: 01/02/2024 18:08:20

Comment:

ID Search Hits

#1 '25 hydroxyvitamin d':ti,ab,kw OR 'alfacalcidol':ti,ab,kw OR 'alphacalcidol':ti,ab,kw OR 'calcitriol':ti,ab,kw OR 'cholecalciferol*':ti,ab,kw OR 'colecalciferol':ti,ab,kw OR 'doxercalciferol':ti,ab,kw OR 'ergocalciferol*':ti,ab,kw OR 'paricalcitol':ti,ab,kw OR 'vitamin d':ti,ab,kw OR 'vitamin d2':ti,ab,kw OR 'vitamin d3':ti,ab,kw OR 'vit D*':ti,ab,kw OR 'vitD*':ti,ab,kw 20491

#2 MeSH descriptor: [Vitamin D] explode all trees 6956

#3 'biphosphonate':ti,ab,kw OR 'biphosphonates':ti,ab,kw OR 'bisphosphonate':ti,ab,kw OR 'bisphosphonate derivative':ti,ab,kw OR 'bisphosphonates':ti,ab,kw OR 'bisphosphonic acid derivative':ti,ab,kw OR 'diphosphonate derivative':ti,ab,kw OR 'diphosphonate series':ti,ab,kw OR 'diphosphonates':ti,ab,kw OR 'diphosphonic acid derivative':ti,ab,kw 3410

#4 MeSH descriptor: [Diphosphonates] explode all trees 3071

#5 'age related bone loss':ti,ab,kw OR 'age related bone losses':ti,ab,kw OR 'age related osteoporoses':ti,ab,kw OR 'age related osteoporosis':ti,ab,kw OR 'bone loss, age related':ti,ab,kw OR 'bone losses, age related':ti,ab,kw OR 'decalcification, pathologic':ti,ab,kw OR 'endocrine osteoporosis':ti,ab,kw OR 'osteoporoses':ti,ab,kw OR 'osteoporoses, age related':ti,ab,kw OR 'osteoporoses, senile':ti,ab,kw OR 'osteoporosis':ti,ab,kw OR 'osteoporosis, age related':ti,ab,kw OR 'osteoporosis, involutional':ti,ab,kw OR 'osteoporosis, post traumatic':ti,ab,kw OR 'osteoporosis, senile':ti,ab,kw OR 'osteoporotic decalcification':ti,ab,kw OR 'pathologic decalcification':ti,ab,kw OR 'post traumatic osteoporoses':ti,ab,kw OR 'post traumatic osteoporosis':ti,ab,kw OR 'senile osteoporoses':ti,ab,kw OR 'senile osteoporosis':ti,ab,kw OR 'involutional osteoporosis':ti,ab,kw OR 'involutional osteoporoses':ti,ab,kw 14807

#6 MeSH descriptor: [Osteoporosis] explode all trees 5792

#7 (#1 OR #2) AND (#3 OR #4) AND (#5 OR #6) 820

**2.Embase**

| No. | Query | Results | Date |
| --- | --- | --- | --- |
| #9 | #8 AND 'Article'/it | 1250 | 1-Feb-24 |
| #8 | (#1 OR #2) AND (#3 OR #4) AND (#5 OR #6) AND #7 | 2408 | 1-Feb-24 |
| #7 | 'random*':ti,ab,kw OR 'control*':ti,ab,kw | 7102772 | 1-Feb-24 |
| #6 | 'osteoporosis'/exp | 154778 | 1-Feb-24 |
| #5 | 'age related bone loss':ti,ab,kw OR 'age related bone losses':ti,ab,kw OR 'age related osteoporoses':ti,ab,kw OR 'age related osteoporosis':ti,ab,kw OR 'bone loss, age related':ti,ab,kw OR 'bone losses, age related':ti,ab,kw OR 'decalcification, pathologic':ti,ab,kw OR 'endocrine osteoporosis':ti,ab,kw OR 'osteoporoses':ti,ab,kw OR 'osteoporoses, age related':ti,ab,kw OR 'osteoporoses, senile':ti,ab,kw OR 'osteoporosis':ti,ab,kw OR 'osteoporosis, age related':ti,ab,kw OR 'osteoporosis, involutional':ti,ab,kw OR 'osteoporosis, post traumatic':ti,ab,kw OR 'osteoporosis, senile':ti,ab,kw OR 'osteoporotic decalcification':ti,ab,kw OR 'pathologic decalcification':ti,ab,kw OR 'post traumatic osteoporoses':ti,ab,kw OR 'post traumatic osteoporosis':ti,ab,kw OR 'senile osteoporoses':ti,ab,kw OR 'senile osteoporosis':ti,ab,kw OR 'involutional osteoporosis':ti,ab,kw OR 'involutional osteoporoses':ti,ab,kw | 134547 | 1-Feb-24 |
| #4 | 'bisphosphonic acid derivative'/exp | 77738 | 1-Feb-24 |
| #3 | 'biphosphonate':ti,ab,kw OR 'biphosphonates':ti,ab,kw OR 'bisphosphonate':ti,ab,kw OR 'bisphosphonate derivative':ti,ab,kw OR 'bisphosphonates':ti,ab,kw OR 'bisphosphonic acid derivative':ti,ab,kw OR 'diphosphonate derivative':ti,ab,kw OR 'diphosphonate series':ti,ab,kw OR 'diphosphonates':ti,ab,kw OR 'diphosphonic acid derivative':ti,ab,kw | 32991 | 1-Feb-24 |
| #2 | 'vitamin d'/exp | 180706 | 1-Feb-24 |
| #1 | '25 hydroxyvitamin d':ti,ab,kw OR 'alfacalcidol':ti,ab,kw OR 'alphacalcidol':ti,ab,kw OR 'calcitriol':ti,ab,kw OR 'cholecalciferol*':ti,ab,kw OR 'colecalciferol':ti,ab,kw OR 'doxercalciferol':ti,ab,kw OR 'ergocalciferol*':ti,ab,kw OR 'paricalcitol':ti,ab,kw OR 'vitamin d':ti,ab,kw OR 'vitamin d2':ti,ab,kw OR 'vitamin d3':ti,ab,kw OR 'vit d*':ti,ab,kw OR 'vitd*':ti,ab,kw | 136392 | 1-Feb-24 |

**3.Pubmed**

| Search number | Query | Filters | Search Details | Results | Time |
| --- | --- | --- | --- | --- | --- |
| 8 | (#1 OR #2) AND (#3 OR #4) AND (#5 OR #6) AND #7 | from 1000/1/1 - 2024/2/1 | (((("25 Hydroxyvitamin D"[Title/Abstract] OR "alfacalcidol"[Title/Abstract] OR "alphacalcidol"[Title/Abstract] OR "calcitriol"[Title/Abstract] OR "cholecalciferol"[Title/Abstract] OR "colecalciferol"[Title/Abstract] OR "doxercalciferol"[Title/Abstract] OR "ergocalciferol"[Title/Abstract] OR "paricalcitol"[Title/Abstract] OR "vitamin D"[Title/Abstract] OR "vitamin D2"[Title/Abstract] OR "vitamin D3"[Title/Abstract] OR "vit d*"[Title/Abstract] OR "vitd*"[Title/Abstract]) AND 1000/01/01:2024/02/01[Date - Publication]) OR (("vitamin D"[MeSH Terms] OR "ergocalciferols"[MeSH Terms]) AND 1000/01/01:2024/02/01[Date - Publication])) AND ((("biphosphonate"[Title/Abstract] OR "biphosphonates"[Title/Abstract] OR "bisphosphonate"[Title/Abstract] OR "bisphosphonate derivative"[Title/Abstract] OR "bisphosphonates"[Title/Abstract] OR "diphosphonate derivative"[Title/Abstract] OR "Diphosphonates"[Title/Abstract]) AND 1000/01/01:2024/02/01[Date - Publication]) OR ("Diphosphonates"[MeSH Terms] AND 1000/01/01:2024/02/01[Date - Publication])) AND ((("Age Related Bone Loss"[Title/Abstract] OR "Age Related Bone Losses"[Title/Abstract] OR "Age Related Osteoporoses"[Title/Abstract] OR "Age Related Osteoporosis"[Title/Abstract] OR "bone loss age related"[Title/Abstract] OR "decalcification pathologic"[Title/Abstract] OR "endocrine osteoporosis"[Title/Abstract] OR "Osteoporoses"[Title/Abstract] OR "Osteoporosis"[Title/Abstract] OR "osteoporosis age related"[Title/Abstract] OR "osteoporosis post traumatic"[Title/Abstract] OR "osteoporosis senile"[Title/Abstract] OR "pathologic decalcification"[Title/Abstract] OR "Post Traumatic Osteoporosis"[Title/Abstract] OR "Senile Osteoporosis"[Title/Abstract] OR "Involutional Osteoporosis"[Title/Abstract]) AND 1000/01/01:2024/02/01[Date - Publication]) OR (("osteoporosis, postmenopausal"[MeSH Terms] OR "Osteoporosis"[MeSH Terms]) AND 1000/01/01:2024/02/01[Date - Publication])) AND (("random*"[Title/Abstract] OR "control*"[Title/Abstract]) AND 1000/01/01:2024/02/01[Date - Publication])) AND (1000/1/1:2024/2/1[pdat]) | 689 | 22:41:55 |
| 7 | "random*"[Title/Abstract] OR "control*"[Title/Abstract] | from 1000/1/1 - 2024/2/1 | ("random*"[Title/Abstract] OR "control*"[Title/Abstract]) AND (1000/1/1:2024/2/1[pdat]) | 5,562,324 | 22:41:15 |
| 6 | Osteoporosis[MeSH Terms] | from 1000/1/1 - 2024/2/1 | ("osteoporosis, postmenopausal"[MeSH Terms] OR "osteoporosis"[MeSH Terms]) AND (1000/1/1:2024/2/1[pdat]) | 64,211 | 22:40:48 |
| 5 | "Age Related Bone Loss"[Title/Abstract] OR "Age Related Bone Losses"[Title/Abstract] OR "Age Related Osteoporoses"[Title/Abstract] OR "Age Related Osteoporosis"[Title/Abstract] OR "bone loss age related"[Title/Abstract] OR "decalcification pathologic"[Title/Abstract] OR "endocrine osteoporosis"[Title/Abstract] OR "Osteoporoses"[Title/Abstract] OR "Osteoporosis"[Title/Abstract] OR "osteoporosis age related"[Title/Abstract] OR "osteoporosis post traumatic"[Title/Abstract] OR "osteoporosis senile"[Title/Abstract] OR "pathologic decalcification"[Title/Abstract] OR "Post Traumatic Osteoporosis"[Title/Abstract] OR "Senile Osteoporosis"[Title/Abstract] OR "Involutional Osteoporosis"[Title/Abstract] | from 1000/1/1 - 2024/2/1 | ("Age Related Bone Loss"[Title/Abstract] OR "Age Related Bone Losses"[Title/Abstract] OR "Age Related Osteoporoses"[Title/Abstract] OR "Age Related Osteoporosis"[Title/Abstract] OR "bone loss age related"[Title/Abstract] OR "decalcification pathologic"[Title/Abstract] OR "endocrine osteoporosis"[Title/Abstract] OR "Osteoporoses"[Title/Abstract] OR "Osteoporosis"[Title/Abstract] OR "osteoporosis age related"[Title/Abstract] OR "osteoporosis post traumatic"[Title/Abstract] OR "osteoporosis senile"[Title/Abstract] OR "pathologic decalcification"[Title/Abstract] OR "Post Traumatic Osteoporosis"[Title/Abstract] OR "Senile Osteoporosis"[Title/Abstract] OR "Involutional Osteoporosis"[Title/Abstract]) AND (1000/1/1:2024/2/1[pdat]) | 87,018 | 22:40:28 |
| 4 | Diphosphonates[MeSH Terms] | from 1000/1/1 - 2024/2/1 | ("diphosphonates"[MeSH Terms]) AND (1000/1/1:2024/2/1[pdat]) | 28,689 | 22:39:34 |
| 3 | "biphosphonate"[Title/Abstract] OR "biphosphonates"[Title/Abstract] OR "bisphosphonate"[Title/Abstract] OR "bisphosphonate derivative"[Title/Abstract] OR "bisphosphonates"[Title/Abstract] OR "diphosphonate derivative"[Title/Abstract] OR "Diphosphonates"[Title/Abstract] | from 1000/1/1 - 2024/2/1 | ("biphosphonate"[Title/Abstract] OR "biphosphonates"[Title/Abstract] OR "bisphosphonate"[Title/Abstract] OR "bisphosphonate derivative"[Title/Abstract] OR "bisphosphonates"[Title/Abstract] OR "diphosphonate derivative"[Title/Abstract] OR "Diphosphonates"[Title/Abstract]) AND (1000/1/1:2024/2/1[pdat]) | 21,273 | 22:39:12 |
| 2 | Vitamin D[MeSH Terms] | from 1000/1/1 - 2024/2/1 | ("vitamin d"[MeSH Terms] OR "ergocalciferols"[MeSH Terms]) AND (1000/1/1:2024/2/1[pdat]) | 70,010 | 22:31:46 |
| 1 | "25 Hydroxyvitamin D"[Title/Abstract] OR "alfacalcidol"[Title/Abstract] OR "alphacalcidol"[Title/Abstract] OR "calcitriol"[Title/Abstract] OR "cholecalciferol"[Title/Abstract] OR "colecalciferol"[Title/Abstract] OR "doxercalciferol"[Title/Abstract] OR "ergocalciferol"[Title/Abstract] OR "paricalcitol"[Title/Abstract] OR "vitamin D"[Title/Abstract] OR "vitamin D2"[Title/Abstract] OR "vitamin D3"[Title/Abstract] OR "vit D*"[Title/Abstract] OR "vitD*"[Title/Abstract] | from 1000/1/1 - 2024/2/1 | ("25 Hydroxyvitamin D"[Title/Abstract] OR "alfacalcidol"[Title/Abstract] OR "alphacalcidol"[Title/Abstract] OR "calcitriol"[Title/Abstract] OR "cholecalciferol"[Title/Abstract] OR "colecalciferol"[Title/Abstract] OR "doxercalciferol"[Title/Abstract] OR "ergocalciferol"[Title/Abstract] OR "paricalcitol"[Title/Abstract] OR "vitamin D"[Title/Abstract] OR "vitamin D2"[Title/Abstract] OR "vitamin D3"[Title/Abstract] OR "vit d*"[Title/Abstract] OR "vitd*"[Title/Abstract]) AND (1000/1/1:2024/2/1[pdat]) | 92,660 | 22:31:29 |

**4.WOS**

| No. | Query | Results |
| --- | --- | --- |
| #1 | (TI =((“25 hydroxyvitamin d”) OR (“alfacalcidol”) OR (“alphacalcidol”) OR (“calcitriol”) OR (“cholecalciferol*”) OR (“colecalciferol”) OR (“doxercalciferol”) OR (“ergocalciferol*”) OR (“paricalcitol”) OR (“vitamin d”) OR (“vitamin d2”) OR (“vitamin d3”) OR (“vit D*”) OR (“vitD*”)) OR AB=((“25 hydroxyvitamin d”) OR (“alfacalcidol”) OR (“alphacalcidol”) OR (“calcitriol”) OR (“cholecalciferol*”) OR (“colecalciferol”) OR (“doxercalciferol”) OR (“ergocalciferol*”) OR (“paricalcitol”) OR (“vitamin d”) OR (“vitamin d2”) OR (“vitamin d3”) OR (“vit D*”) OR (“vitD*”)) OR AK=((“25 hydroxyvitamin d”) OR (“alfacalcidol”) OR (“alphacalcidol”) OR (“calcitriol”) OR (“cholecalciferol*”) OR (“colecalciferol”) OR (“doxercalciferol”) OR (“ergocalciferol*”) OR (“paricalcitol”) OR (“vitamin d”) OR (“vitamin d2”) OR (“vitamin d3”) OR (“vit D*”) OR (“vitD*”))) AND (TI=((“age related bone loss”) OR (“age related bone losses”) OR (“age related osteoporoses”) OR (“age related osteoporosis”) OR (“bone loss, age related”) OR (“bone losses, age related”) OR (“decalcification, pathologic”) OR (“endocrine osteoporosis”) OR (“osteoporoses”) OR (“osteoporoses, age related”) OR (“osteoporoses, senile”) OR (“osteoporosis”) OR (“osteoporosis, age related”) OR (“osteoporosis, involutional”) OR (“osteoporosis, post traumatic”) OR (“osteoporosis, senile”) OR (“osteoporotic decalcification”) OR (“pathologic decalcification”) OR (“post traumatic osteoporoses”) OR (“post traumatic osteoporosis”) OR (“senile osteoporoses”) OR (“senile osteoporosis”) OR (“involutional osteoporosis”) OR (“involutional osteoporosis”)) OR AB=((“age related bone loss”) OR (“age related bone losses”) OR (“age related osteoporoses”) OR (“age related osteoporosis”) OR (“bone loss, age related”) OR (“bone losses, age related”) OR (“decalcification, pathologic”) OR (“endocrine osteoporosis”) OR (“osteoporoses”) OR (“osteoporoses, age related”) OR (“osteoporoses, senile”) OR (“osteoporosis”) OR (“osteoporosis, age related”) OR (“osteoporosis, involutional”) OR (“osteoporosis, post traumatic”) OR (“osteoporosis, senile”) OR (“osteoporotic decalcification”) OR (“pathologic decalcification”) OR (“post traumatic osteoporoses”) OR (“post traumatic osteoporosis”) OR (“senile osteoporoses”) OR (“senile osteoporosis”) OR (“involutional osteoporosis”) OR (“involutional osteoporosis”)) OR AK=((“age related bone loss”) OR (“age related bone losses”) OR (“age related osteoporoses”) OR (“age related osteoporosis”) OR (“bone loss, age related”) OR (“bone losses, age related”) OR (“decalcification, pathologic”) OR (“endocrine osteoporosis”) OR (“osteoporoses”) OR (“osteoporoses, age related”) OR (“osteoporoses, senile”) OR (“osteoporosis”) OR (“osteoporosis, age related”) OR (“osteoporosis, involutional”) OR (“osteoporosis, post traumatic”) OR (“osteoporosis, senile”) OR (“osteoporotic decalcification”) OR (“pathologic decalcification”) OR (“post traumatic osteoporoses”) OR (“post traumatic osteoporosis”) OR (“senile osteoporoses”) OR (“senile osteoporosis”) OR (“involutional osteoporosis”) OR (“involutional osteoporosis”))) AND (TI=((“biphosphonate”) OR (“biphosphonates”) OR (“bisphosphonate”) OR (“bisphosphonate derivative”) OR (“bisphosphonates”) OR (“bisphosphonic acid derivative”) OR (“diphosphonate derivative”) OR (“diphosphonate series”) OR (“diphosphonates”) OR (“diphosphonic acid derivative”)) OR AB=((“biphosphonate”) OR (“biphosphonates”) OR (“bisphosphonate”) OR (“bisphosphonate derivative”) OR (“bisphosphonates”) OR (“bisphosphonic acid derivative”) OR (“diphosphonate derivative”) OR (“diphosphonate series”) OR (“diphosphonates”) OR (“diphosphonic acid derivative”)) OR AK=((“biphosphonate”) OR (“biphosphonates”) OR (“bisphosphonate”) OR (“bisphosphonate derivative”) OR (“bisphosphonates”) OR (“bisphosphonic acid derivative”) OR (“diphosphonate derivative”) OR (“diphosphonate series”) OR (“diphosphonates”) OR (“diphosphonic acid derivative”))) AND (TI=((“random*”) OR (control*)) OR AB=((“random*”) OR (control*)) OR AK=((“random*”) OR (control*))) | 510 |
